# Dendritic cell maturation, but not type I interferon exposure, restricts infection by HTLV-1, and viral transmission to T-cells

**DOI:** 10.1371/journal.ppat.1006353

**Published:** 2017-04-20

**Authors:** Gergès Rizkallah, Sandrine Alais, Nicolas Futsch, Yuetsu Tanaka, Chloé Journo, Renaud Mahieux, Hélène Dutartre

**Affiliations:** 1 International Center for Research in Infectiology, Retroviral Oncogenesis laboratory, INSERM U1111 –Université Claude Bernard Lyon 1, CNRS, UMR5308, Ecole Normale Supérieure de Lyon, Université Lyon, Lyon, France; 2 Equipe labellisée “Ligue Nationale Contre le Cancer”, Lyon, France; 3 Department of Immunology, Graduate School of Medicine, University of the Ryukyus, Uehara 207, Nishihara-cho, Okinawa, Japan; Miller School of Medicine, UNITED STATES

## Abstract

Human T lymphotropic Virus type 1 (HTLV-1) is the etiological agent of Adult T cell Leukemia/Lymphoma (ATLL) and HTLV-1-Associated Myelopathy/Tropical Spastic Paraparesis (HAM/TSP). Both CD4^+^ T-cells and dendritic cells (DCs) infected with HTLV-1 are found in peripheral blood from HTLV-1 carriers. We previously demonstrated that monocyte-derived IL-4 DCs are more susceptible to HTLV-1 infection than autologous primary T-cells, suggesting that DC infection precedes T-cell infection. However, during blood transmission, breast-feeding or sexual transmission, HTLV-1 may encounter different DC subsets present in the blood, the intestinal or genital mucosa respectively. These different contacts may impact HTLV-1 ability to infect DCs and its subsequent transfer to T-cells. Using *in vitro* monocyte-derived IL-4 DCs, TGF-β DCs and IFN-α DCs that mimic DCs contacting HTLV-1 *in vivo*, we show here that despite their increased ability to capture HTLV-1 virions, IFN-α DCs restrict HTLV-1 productive infection. Surprisingly, we then demonstrate that it is not due to the antiviral activity of type–I interferon produced by IFN-α DCs, but that it is likely to be linked to a distinct trafficking route of HTLV-1 in IL-4 DCs vs. IFN-α DCs. Finally, we demonstrate that, in contrast to IL-4 DCs, IFN-α DCs are impaired in their capacity to transfer HTLV-1 to CD4 T-cells, both after viral capture and trans-infection and after their productive infection. In conclusion, the nature of the DCs encountered by HTLV-1 upon primo-infection and the viral trafficking route through the vesicular pathway of these cells determine the efficiency of viral transmission to T-cells, which may condition the fate of infection.

## Author summary

Human T-Lymphotropic Virus type 1 (HTLV-1) is the etiological agent of Adult T-cell Leukemia/Lymphoma (ATLL) and HTLV-1-Associated Myelopathy/Tropical Spastic Paraparesis (HAM/TSP). In chronically infected patients, the provirus is mainly detected in the CD4^+^ T-cell population. However, beside lymphocytes, HTLV-1 infects blood or monocyte-derived dendritic cells (DCs) *in vitro*. Moreover, we previously showed that DCs are more susceptible to HTLV-1 infection than autologous T-cells, suggesting that DCs might be the first cells to be infected upon primo-infection, and important intermediaries for the viral spread to surrounding lymphocytes. Interestingly, different DC subsets are found in the blood or in the mucosa, the two entry routes used by HTLV-1 during infection of new individuals. In this study, we show for the first time that the different DC subsets are not equally susceptible to HTLV-1 infection. Furthermore we demonstrate that DC subsets with an increased ability to capture HTLV-1 virion are restricted to HTLV-1 productive infection. This restriction is linked to DC maturation but not to the antiviral activity of IFN-α produced by resistant DCs. Finally, we demonstrate that efficient viral transmission to T-cells is dependent upon DC productive infection rather than trans-infection. Altogether, our results indicate that the nature of the DCs encountered by HTLV-1 upon primo-infection determines the efficiency of viral transmission to T-cells, which may condition the fate of infection.

## Introduction

Human T-Lymphotropic Virus type 1 (HTLV-1) infects 5–10 million people [1]. HTLV-1 is mainly present in Japan, inter-tropical Africa, the Caribbean and South America [2,3]. After a long period of clinical latency, HTLV-1 infection leads, in a fraction of infected individuals, either to Adult T-cell Leukemia/Lymphoma (ATLL) [4], an uncontrolled CD4^+^ T–cell proliferation of very poor prognosis, or to an inflammatory disorder named HTLV-1 Associated Myelopathy / Tropical Spastic Paraparesis (HAM/TSP) [5]. *In vivo*, in chronically infected individuals, HTLV-1 is mainly found in CD4^+^ T-cells, but infected dendritic cells (DCs) are also detected *in vitro* and more importantly *in vivo* [6,7,8,9,10,11]. Their function is subsequently altered *in vivo* [11,12,13] and these cells are therefore likely to be involved in viral pathogenesis.

The role of DC infection in HTLV-1 dissemination to T-cells has been investigated in mice exposed to chimeric HTLV-1-infected cells, in which the HTLV-1 envelope had been replaced by that of the Moloney murine leukemia virus, to allow HTLV-1 to enter murine cells. In this context, DC depletion leads to a decreased proviral load in mouse CD4^+^ T-cells [14]. In addition, HTLV-1 viruses harboring mutations in the p12 and p30 regulatory genes that have lost their ability to infect human DCs, also have an impaired ability to infect macaques [15]. Altogether, these *in vivo* experiments strongly suggest that infection of DCs is required for the establishment and maintenance of HTLV-1 infection in animal models. Consistent with these data, we recently showed that human monocytes-derived dendritic cells (MDDCs) are more susceptible to HTLV-1 infection than autologous lymphocytes *in vitro* [9], supporting a model where DC infection represents an important step upon primo-infection *in vivo*.

In addition to the blood, DCs are widely distributed in peripheral tissues and in mucosae, where antigen capture might occur. DCs play a central role in activating naïve T-cells and directing the subsequent immune response. DCs are constituted of several distinct subsets that differ in their ability to activate immunity or to promote tolerance [16]. Since HTLV-1 is transmitted through contact with infected cells present in maternal milk, semen or blood, viruses may first be in contact with different DC subsets present in these different tissues. The interaction of HTLV-1-infected cells with a given DC subset may thus affect the subsequent immune response. In addition, interaction with distinct DC subsets might have an impact on the risk of developing ATLL in the case of mother-to-child transmission or on the risk of developing HAM/TSP in the case of infection with contaminated blood [17,18].

We therefore investigated whether different MDDC subsets that are similar to tissue-resident DCs exposed to HTLV-1 during primary infection in humans, are equally susceptible to HTLV-1 infection. Human MDDCs were generated with cytokines that allow differentiation into different subsets: interleukin-4 (IL-4) for myeloid DCs of the blood, transforming growth factor beta (TGF-β) for mucosal DCs of the gut and interferon-alpha (IFN-α) for inflammatory DCs found in injured skin [19]. We demonstrate that IL-4 DCs and to a lesser extent TGF-β DCs are susceptible to HTLV-1 infection, while IFN-α DCs are not. We then demonstrate that IFN-α DC resistance to HTLV-1 infection is not due to IFN-α production. In contrast, we demonstrate that DC maturation, in addition to viral trafficking through acidic vesicles, contributes to DC resistance to HTLV-1 infection. Finally, we show that HTLV-1 productive infection of immature DCs, rather than viral capture by matured-DCs, is required for HTLV-1 transmission from DCs to T-cells. These results demonstrate for the first time that immature DCs represent the main DC population that allows infection of T-cells.

## Material and methods

### Ethics statement

The French Comité de protection des personnes Ile de France has approved the use of human samples used in this study. CPP-IDF-2012-10-04SC to Dr. Gessain (Institut Pasteur, France).

### Reagents and antibodies

Chlorpromazine (20 μM, resuspended in H_2_O), diphenyl iodonium chloride (10 μM, resuspended in DMSO); dynasore (100 μM, resuspended in DMSO), methyl-β-cyclodextrin (10 mM, resuspended in H_2_O), nystatin (270 μM, resuspended in DMSO), amiloride (375 μM, resuspended in H_2_O), latrunculin (2 μM, resuspended in DMSO), cytochalasin D (2 μM, resuspended in DMSO), genistein (200 μM, resuspended in MeOH), wortmanin (40 μM, resuspended in DMSO), rottlerin (25 μM, resuspended in EtOH), Gö6953 (1 μM, resuspended in DMSO), NSC23766 trihydrochloride (250 μM, resuspended in H_2_O) and IPA-3 (125 μM, resuspended in DMSO) were purchased from Sigma.

Toll like receptor (TLR)-2 agonist (PAM3CSK4; 1 μg/ml); TLR-5 agonist (Flagellin; 200 ng/ml); TLR-3 agonist (Poly(I:C); 10 μg/ml); TLR-4 agonist (LPS; 1 μg/ml); TLR-7/8 agonist (R848; 3 μg/ml) were purchased from Invivogen. Recombinant IFN-α (500–2000 IU/ml) was purchased from Tebu-Bio.

The following antibodies were purchased from BD Biosciences: APC-H7-labeled mouse anti-Human CD14 (MφP9); V450-coupled mouse anti-Human CD11c (clone Ly6); PE-Cy7-coupled mouse anti-Human CD40 (clone 5C3); PerCPCy5.5-coupled mouse anti-Human HLA-DR (clone G46.6). APC-coupled mouse anti-Human DC-SIGN (DCN47.5) and Vio-Bright-FITC-coupled mouse anti-Human BDCA4 (clone AD517F6) were from Miltenyi. PE-coupled mouse anti-Human CD86 (clone IT2.2) was from Ebioscience. Mouse anti-HTLV-1 Gag p19 antibody (clone TP7) was from Zeptometrik, and biotin-coupled mouse anti-HTLV-1 Tax antibody (clone LT4, [20]) was provided by Pr Tanaka.

### Cells

Jurkat cells stably transfected with a plasmid encoding the luciferase (Luc) gene under the control of the HTLV-1 long terminal repeat (LTR) promotor (Jurkat-LTR-Luc) [21] were maintained under hygromycin (450 μg/ml, Sigma) selection in RPMI 1640 medium supplemented with 10% fetal calf serum (FCS; Gibco Life Technologies) and penicillin-streptomycin (100 μg/ml; Gibco Life Technologies). HTLV-1 infected T-cells (C91-PL) were maintained in RPMI 1640 medium supplemented with 10% FCS and penicillin-streptomycin (100 μg/ml). The human fibrosarcoma cell line containing a plasmid encoding the luciferase gene under the control of the immediate early IFN inducible 6–16 promoter (HL116) [22] were maintained under HAT selection in DMEM medium supplemented with 10% FCS and penicillin-streptomycin (100 μg/ml). Monocyte-derived dendritic cells were maintained in complete MDDC medium composed of RPMI 1640 medium supplemented with 10% FCS, 100 μg/ml of penicillin-streptomycin, non-essential amino acids (2.5 mM; Gibco Life Technologies), sodium pyruvate (1 mM; Sigma), β-mercaptoethanol (0.05 mM; Gibco Life Technologies), and HEPES (10 mM; Gibco Life Technologies). Granulocyte-macrophage colony stimulating factor (GM-CSF; 100 ng/ml; Miltenyi) and interleukine 4 (IL-4, 100 ng/ml; Miltenyi), or transforming growth factor beta (TGF-β, 10 ng/ml, Eurobio), or interferon alpha (IFN-α, 500 IU/ml, Tebu-bio) were added to the medium for differentiation. All cells were grown at 37°C in 5% CO_2_.

### Human primary monocyte-derived dendritic cells (MDDCs)

Blood was collected by Etablissement Français du Sang (Lyon, France) from non-infected blood donors. Peripheral blood mononuclear cells (PBMCs) were isolated from heparinized blood on Ficoll density gradient. Monocytes were purified from PBMCs on Percoll gradients. Cells were cryopreserved in 10% dimethylsulfoxide (DMSO) - 50% FCS—40% culture medium before being used. Dendritic cells were generated after culture of monocytes in complete MDDC medium. IL-4 DCs were obtained after culture of monocytes in complete MDDC medium supplemented with GM-CSF and IL-4 for 5 days. Fresh cytokines were added 72 h later. When indicated, IL-4 DCs were further treated for 18 h by the addition of TLR agonists or recombinant IFN-α in the MDDC medium. TGF-β DCs were obtained after culture of monocytes in complete MDDC medium supplemented with GM-CSF, IL-4 and TGF-β for 5 days. Fresh cytokines were added 72 h later. IFN-α DCs were obtained from monocytes cultured in complete MDDC medium supplemented with GM-CSF and IFN-α for 3 days. Fresh cytokines were added 24 h later.

### MDDC infection

MDDCs (2.5 x 10^5^ cells) were co-cultured with C91-PL cells (2.5 x 10^4^ cells), pre-treated with mitomycin C (50 μg/ml, Sigma). MDDCs were then collected after 3h or 72h of co-culture, fixed and used for flow cytometry analyses (FACS Canto II; BD Biosciences) and imaging. When indicated, MDDCs were infected in presence of azido-thymidine (AZT, 10 μg/ml, Sigma) or treated with chloroquine (150 μM, Sigma) or diphenyl iodonium chloride (10 μM, Sigma) before co-culture with mitomycin-treated C91-PL cells.

When indicated, MDDCs (2.5 x 10^5^ cells) were infected by biofilm preparation (100 μl) purified as previously described [9]. At each time point, MDDCs were collected, washed several times with PBS and stored as pellets before genomic DNA extraction and real-time PCR analysis. When indicated, biofilm was heat-inactivated at 56°C before contact with MDDCs.

### HTLV-1 capture assay

MDDCs (2.5 x 10^5^ cells) were plated in 48-well plates and treated with drugs targeting several endocytosis pathways for 3 h. Untreated cells were incubated in the same conditions and were used as controls. Treated and control MDDCs were then co-cultured with mitomycin-treated C91-PL cells (2.5 x 10^4^ cells) for an additional 3 h. Cells were then fixed and stained for flow cytometry analyses.

### Immunofluorescence and confocal microscopy

MDDCs (150 x 10^3^ cells per well) were plated in Lab-Tek chamber slides (Nunc) previously treated with Poly-L-lysine (Sigma, P4832) according to the manufacturer’s instructions and cultured for 18 h. When needed, LPS (1 μg/ml) was added. MDDCs were then co-cultured with mitomycin-treated C91-PL cells (ratio 10:1) for 3 h and fixed with 4% paraformaldehyde (PFA). Cells were extensively washed with PBS, quenched with NH_4_Cl solution (50 mM) and permeabilized with PBS—1% BSA—0.1% saponin. Cells were then stained using a serum from an HTLV-1 infected patient (gift from Dr Gessain, Institut Pasteur) followed by a DyLight 488-labeled Goat anti-Human IgG Antibody (1/1000; Vector laboratories) and anti-CD82 antibody (AM26701PU-N, Acris) followed by a DyLight 549-labeled anti-Mouse IgG Antibody (1/1000; Vector laboratories). When indicated, cells were stained with anti p19^Gag^ antibody followed by a DyLigh 549-labelled anti-mouse antibody and anti-human EE1A antibody (N19, Santa-Cruz biotechnology) followed by a FITC-labelled anti-goat antibody (Dako). After washes in PBS and lastly in water, slides were mounted in Dapi-Fluoromount G (Southern Biotech). Samples were examined under a Leica spectral SP5 confocal microscope equipped with a 63x 1.4–0.6 oil-immersion objective using the LAS-AF software, or using a Zeiss LSM800 confocal microscope equipped with a 63x 1.4 oil immersion objective on the ZEN software. Images were analyzed using ImageJ.

### Determination of the pH of vesicles and quantification of lipid droplets

MDDCs were plated in Lab-Tek wells and incubated with the lysotracker Red DND-99 (5 μM, Thermofisher scientific, L7528) for 30 minutes. Cells were then extensively washed and fixed in 4% PFA. For lipid droplets staining, cells were fixed in 4% PFA, and stained with Nile Red (0.25 mg/ml; Sigma) for 15 min. After several washes in PBS and lastly in water, slides were mounted in Dapi-Fluoromount G and fluorescence analyzed with routine inverted Zeiss axiovert 135 microscope. Fluorescent signals were quantified with ImageJ.

### Flow cytometry

MDDCs (2.5 x 10^5^ cells) were collected, washed in PBS and stained with the following panel: APC-H7-labeled mouse anti-Human CD14; V450-coupled mouse anti-Human CD11c; PE-Cy7-coupled mouse anti-Human CD40; PerCPCy5.5-coupled mouse anti-Human HLA-DR, APC-coupled mouse anti-Human DC-SIGN, Vio-Bright-FITC-coupled mouse anti-Human BDCA4 and PE-coupled mouse anti-Human CD86. To analyze HTLV-1 capture, MDDCs were collected after co-culture with mitomycin-treated C91-PL cells, washed in PBS and fixed with 4% PFA. Cells were permeabilized with PBS—1% BSA—0.1% saponin, and stained with a mouse anti-p19^gag^ antibody (1:1000) followed by a DyLight 488-coupled goat anti-mouse antibody (1:1000). Cells were then washed twice with PBS—1% BSA—0.1% saponin and once with PBS—1% BSA and finally surface-stained with V450-coupled anti-human CD11c antibody.

To analyze productive infection, MDDCs were collected after co-culture with C91-PL, washed in PBS and in normal goat serum (7%, Sigma), fixed and permeabilized according to the manufacturer’s instructions (eBiosciences). Cells were stained with biotin-coupled anti-Tax antibody (LT4) followed by streptavidin labeled with PE-Cy7 (BioLegend, Ozyme). After extensive washes, cells were finally surface-stained with a V450-coupled anti-CD11c antibody. All results were acquired by flow cytometry using at least 50 000 events (FACSCantoII; BD-Biosciences) and analyzed with FlowJo software.

### Genomic DNA extraction and real-time PCR

Genomic DNA was extracted using the Nucleospin blood kit (Macherey-Nagel, Düren, Germany) according to the manufacturer’s instructions. DNA concentration was determined with a NanoDropND1000 spectrophotometer (Thermo Scientific). Real-time quantitative PCR (qPCR) was performed on 10 ng of genomic DNA as previously described [9].

### RNA extraction and RT-PCR

IL-4 DCs (2.5 x 10^5^ cells) were plated in 48-well plates and treated with 500 IU/ml of recombinant IFN-α for 18 h. Cells were washed twice in phosphate-buffered saline (PBS) and total RNA was extracted using the RNeasy minikit (Quiagen, 74106). Extracted RNA was treated with RQ1-RNAse-free DNAse (Promega). RNA concentration was determined with a NanoDropND1000 spectrophotometer (Thermo Scientific). RT-PCR was performed on 500 ng of RNA using the SuperScript Reverse Transcriptase (Thermofisher). cDNA concentration was then determined with a NanoDropND1000 spectrophotometer (Thermo Scientific). cDNA amplification was performed on 200 ng of cDNA using the GoTaq polymerase (Promega) and Mx1 primers as previously described [23]. PCR products were analyzed by electrophoresis on a 2% agarose gel.

### Viral transfer to T-cells

To determine the ability of MDDCs to transfer HTLV-1 without being infected, MDDCs (3 x 10^6^ cells) were exposed to mitomycin-treated C91-PL cells (6 x 10^5^ cells) for 4 h in 6-well plates. Then, DCs were magnetically isolated using the CD304 (BDCA-4/Neuropilin-1) Microbead kit (Miltenyi Biotec, 130-090-532). Isolated DCs (10^5^ cells) were co-cultured with Jurkat-LTR-Luc (10^5^ cells) in 48-well plates. Co-culture was performed for 48 h. Cells were then collected, and luciferase reporter activity assayed (Luciferase kit Promega). Results were normalized according to the amount of proteins determined by Bradford (Biorad).

To determine the ability of productively infected MDDCs to transfer the virus to T-cells, MDDCs (3 x 10^6^ cells) were exposed to mitomycin-treated C91-PL cells (6 x 10^5^ cells) for 4 h in 6-well plates. Then, DCs were magnetically isolated using the CD304 (BDCA-4/Neuropilin-1) Microbead kit. Isolated DCs (1 x 10^5^ cells) were cultured in 48-well plates for 72 hours in the presence or absence of 100 μM of AZT added in culture medium each day. Jurkat-LTR-Luc cells (10^5^ cells) were then added and the co-culture was maintained for 48 h. Cells were then collected, and luciferase reporter activity assayed. Results were normalized according to the amount of proteins determined by Bradford (Biorad).

### Quantification of cell-cell contacts

Jurkat LTR-Luc cells were transduced with Lenti-IRES-GFP as previously described [24]. C91-PL cells were stained with 20 nM of cell tracker red CMPTX (Invitrogen C33542) in RPMI 1640 medium for 30 minutes at 37°C, and washed twice with RPMI 1640 medium supplemented with 10% FCS and penicillin-streptomycin (100 μg/ml) before use. Mock treated and LPS-treated IL-4 DCs or IFN-α DCs (5 x 10^4^ cells) from the same donor were mixed with transduced Jurkat LTR-Luc cells (5 x 10^4^ cells) and red-labelled C91-PL (5 x 10^3^ cells), plated in Lab-Tek chamber slides previously treated with Poly-L-lysine and cultured for 45 minutes at 37°C. Cells were then fixed with 4% PFA for 15 minutes at room temperature. After several washes in PBS, slides were mounted in Dapi-Fluoromount G. Signals were acquired with an Axioimager Z1 microscope (Zeiss), and analyzed with ImageJ. Contacts between unstained MDDCs and GFP transduced Jurkat-LTR-Luc cells were manually counted from at least 10 pictures taken from the same slide.

### Type I interferon quantification

HL116 cells were seeded at 2 x 10^4^ cells/well in 96-U-bottom-well plates and incubated for 24 h. Supernatant collected from MDDCs cultures (100 μl) or serial dilutions of recombinant IFN-α used for standard curve determination were added for an additional 17 h. Cells were then lysed and luciferase activity assayed.

### Statistics and representation of data

One-way analysis of variance (ANOVA) with Bonferroni’s post-hoc multiple comparison test was used to determine statistically significant differences. Paired two-tail t-test was used to compare two groups from the same donor. Differences were considered significant if the p-value was < 0.05.

## Results

### Immature IL-4 DCs and tolerogenic TGF-β DCs but not IFN-α DCs are susceptible to HTLV-1

**Fig 1:**
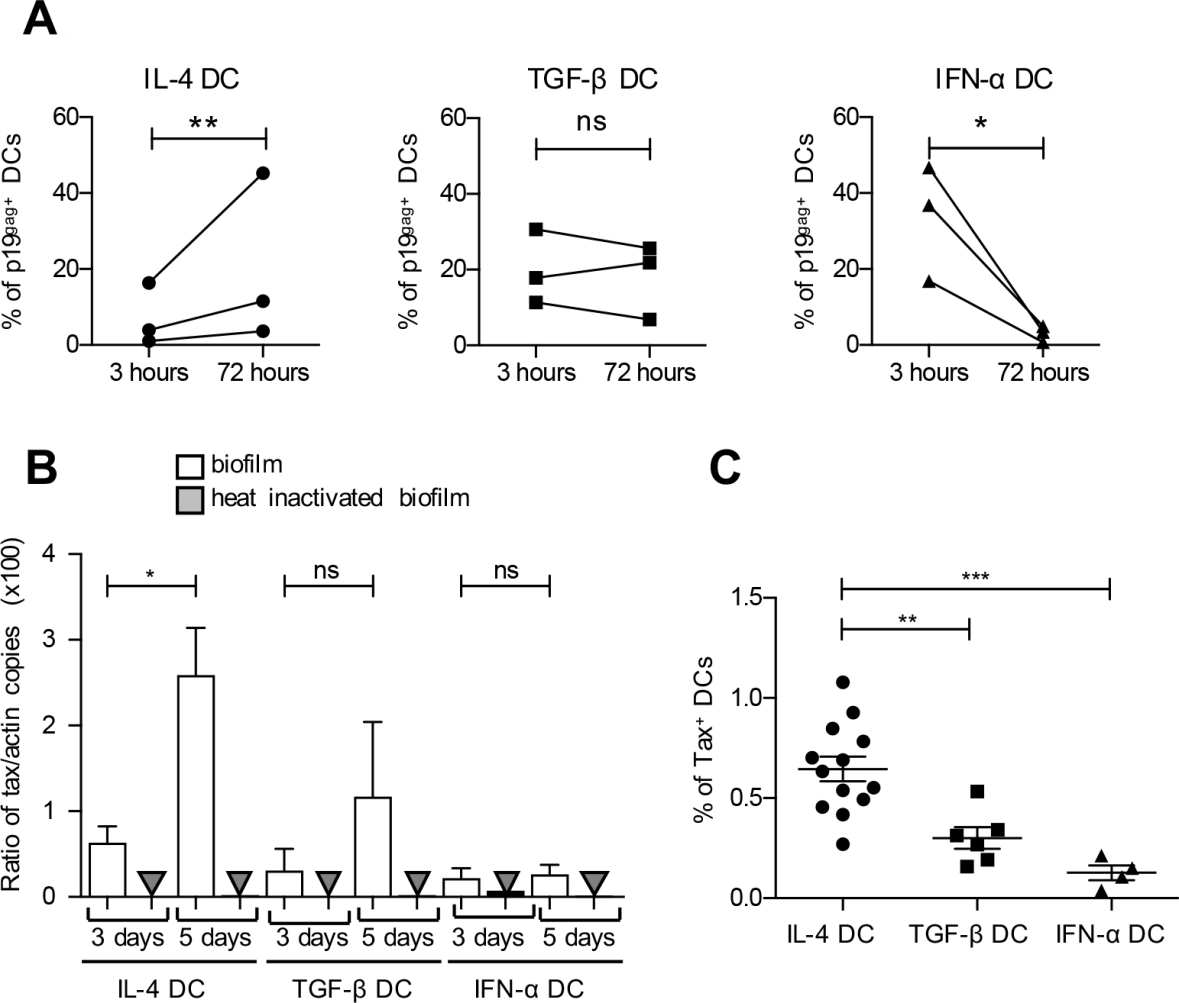
IL-4 DCs, TGF-β DCs and IFN-α DCs are not equally susceptible to HTLV-1 infection. **(A)** MDDCs were co-cultured with mitomycin-treated C91-PL cells, and the percentage of MDDCs expressing the viral p19^gag^ protein was assessed by flow cytometry after 3 h to measure viral capture or 72 h to measure *de novo* viral expression by productively infected MDDCs. MDDCs were discriminated from C91-PL cells by CD11c gating. The results obtained from MDDCs differentiated from the same blood donor are shown as percentage of p19^gag^ protein present in DCs during the time course. Results for 3 independent experiments performed on different donors are shown. Asterisks indicate statistically significant differences calculated using a paired t-test: **p<0.01; *p<0.05; ns: non significant. **(B)** MDDCs were exposed to purified viral biofilm or heat-inactivated biofilm. Genomic DNA was extracted 3 days or 5 days post-exposure and analyzed by real-time PCR. Results are presented as the ratio of *tax/actin* obtained from 3 independent experiments performed on different donors. Mean values are indicated. Asterisks indicate statistically significant differences calculated using a t-test: *p<0.05; ns: non significant. **(C)** MDDCs were co-cultured with mitomycin-treated C91-PL cells for 3 days and the percentage of MDDCs expressing the viral Tax protein determined by flow cytometry. The mean value of at least 4 independent experiments obtained with different blood donors is indicated. Asterisks indicate statistically significant differences calculated using one-way ANOVA followed by Bonferroni’s post-hoc test: ***p<0,001; **p<0.01.

To determine whether all DC subsets were equally susceptible to HTLV-1, IL-4 DCs, TGF-β DCs and IFN-α DCs were generated from human primary monocytes. DC differentiation was controlled by the loss of CD14 expression and the acquisition of CD11c expression (S1A Fig). Consistent with previous data [19], IL-4 DCs and TGF-β DCs displayed the most immature phenotype, with a low surface expression of CD86, CD40 and HLA-DR markers (S1A Fig). Expression of these maturation markers was increased in IFN-α DCs confirming their more mature phenotype (S1A Fig). The three different DC subtypes were then exposed to infected C91-PL cells. Infection was first monitored by the accumulation of the structural p19^gag^ viral protein over time (Fig 1A). In IL-4 DCs, the relative abundance of p19^gag^-positive cells significantly increased between 3h and 72 h post infection (p.i.), while it significantly decreased in IFN-α DCs, suggesting that IFN-α DC are not productively infected. Of note, in TGF-β DCs, the amount of p19^gag^-positive cells did not change significantly, suggesting a reduced level of infection. To confirm this result, the amount of proviral DNA was quantified 3 and 5 days after contact of the different DCs with purified HTLV-1 viral biofilm (Fig 1B). Viral biofilm are membrane-bound viruses that are embedded in extracellular matrix-rich structures at the surface of infected cells [21]. Infection with purified viral biofilm allows quantification of viral DNA resulting from *de novo* infection of target cells, without contamination with viral DNA from C91-PL that occurs when infection is perfomed through co-culture with infected cells. As previously shown [9], proviral DNA was detected in IL-4 DCs exposed to viral biofilm, and its amount significantly increased during the course of infection (Fig 1B). Proviral DNA was also detected in TGF-β DCs exposed to viral biofilm but, consistent with the low accumulation of viral p19^gag^, its amount did not significantly increase during the course of exposure (Fig 1B), suggesting that TGF-β DCs are less susceptible than IL-4 DCs to HTLV-1 infection. In contrast, HTLV-1 viral DNA was barely detected in IFN-α DCs exposed to viral biofilm and its amount did not change during the course of infection (Fig 1B). As a control, no viral DNA was detected when heat-inactivated viral biofilm was used (Fig 1B).

Productive infection was further confirmed by detection of Tax expression, since this viral protein is absent from the viral particle. Consistent with the amount of viral DNA, a higher percentage of IL-4 DCs expressed Tax compared to TGF-β DCs, with a mean of 0,6% infected cells (Fig 1C). A significant reduction in the number of Tax-expressing cells was observed among IL-4 DCs when infection was performed in the presence of AZT (S1B Fig), confirming that Tax expression results from productive infection [9]. In addition, a very low percentage of Tax-expressing cells were found in the IFN-α DC population (Fig 1C) with a mean of 0,1% infected cells. Altogether, these results demonstrate that the IL-4 DC population is more susceptible to HTLV-1 infection than the TGF-β one, and that the IFN-α DC population is resistant to HTLV-1 infection.

### HTLV-1 viral entry is more efficient in DCs that restrict HTLV-1 infection

**Fig 2:**
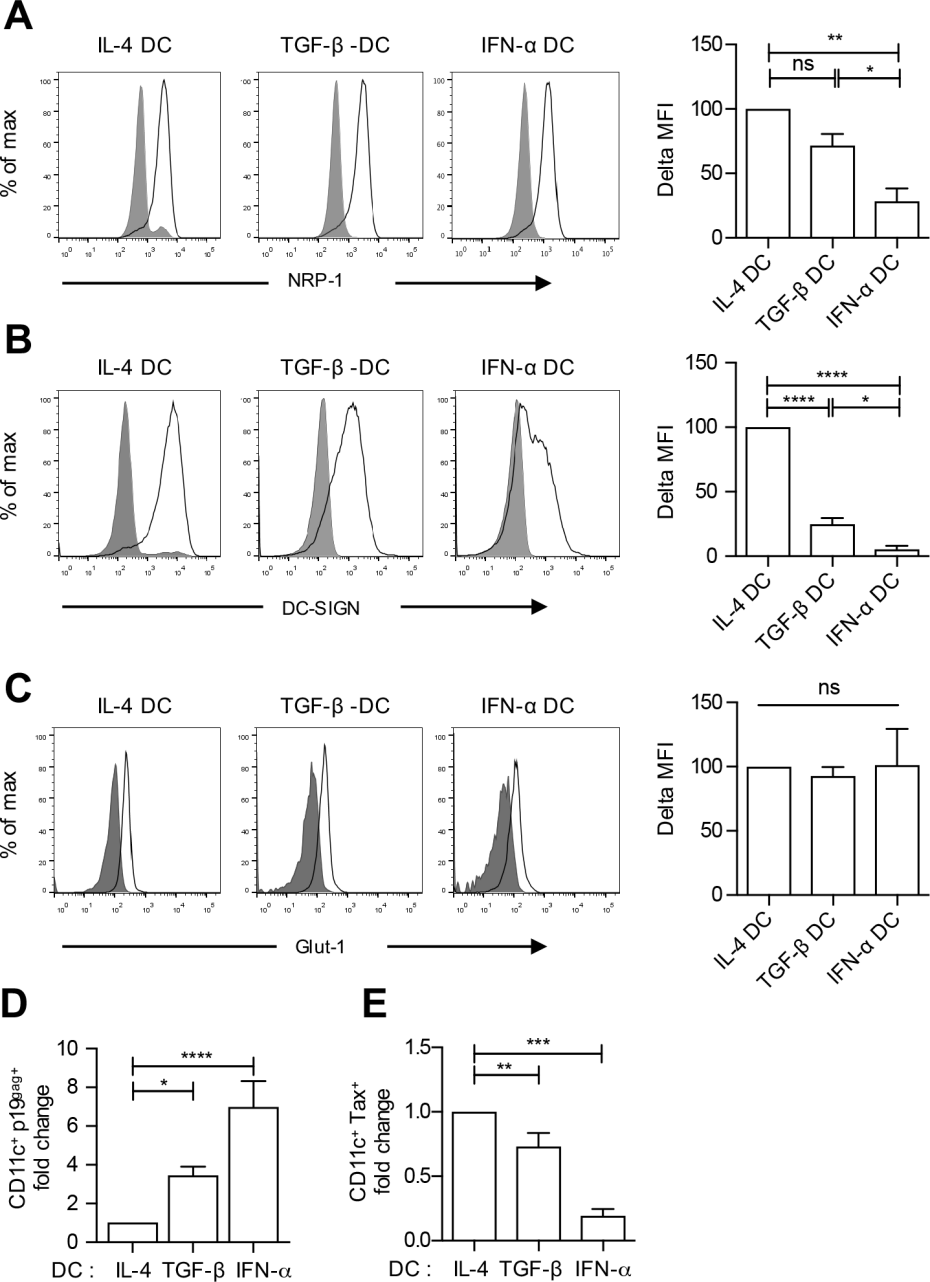
Expression of HTLV-1 receptors in IL-4 DCs, TGF-β DCs and IFN-α DCs is inversely correlated to HTLV-1 capture, but correlated to HTLV-1 productive infection. **(A-C)** Cells were labeled with fluorochrome-coupled antibodies (black lines) directed against **(A)** NRP-1, **(B)** DC-SIGN and **(C)** Glut-1. Cells stained with matched isotype antibodies were used as negative controls (solid grey graphs). Quantification of the mean fluorescence intensity is represented on the right of each panel as the Δ mean fluorescence intensity detected in stained versus unstained cells. Results obtained in 3 independent experiments performed on 3 different donors are presented as percentage of receptor expression normalized to the expression detected in IL-4 DCs. **(D)** IL-4, TGF-β and IFN-α DCs were co-cultured with mitomycin-treated C91-PL cells for 3 h, and the percentage of MDDCs expressing the viral p19^gag^ protein was assessed by flow cytometry. The mean values from at least 4 independent experiments obtained with different donors were normalized to the percentage of p19^gag^ protein detection in IL-4 DCs, and presented as fold change. **(E)** IL-4, TGF-β and IFN-α DCs from the same blood donors were co-cultured with mitomycin-treated C91-PL cells for 3 days and productive infection assessed by flow cytometry allowing viral Tax detection. The number of DCs expressing the viral Tax protein was determined and normalized to the number detected in IL-4 DCs. Results are presented as fold changes. **(A-E)** Asterisks indicate statistically significant differences calculated using one-way ANOVA followed by Bonferroni’s multiple comparison test: ****p<0.0001; *** p<0.001; **p<0.01; *p<0.05; ns: non significant.

To understand the mechanism leading to differential susceptibility to HTLV-1 infection in the three DC subsets, we first controlled the expression of HTLV-1 (Fig 2). Two proteins, the binding receptor NRP-1 and the fusion receptor Glut-1, have been involved in HTLV-1 entry in CD4^+^ T lymphocytes [25]. In addition, DC-SIGN is involved in HTLV-1 binding to DCs [7] and is important for DC infection [6]. NRP-1 was expressed in all DC subsets, although its level was significantly higher in IL-4 and TGF-β DCs than in IFN-α DCs (Fig 2A). Similarly, DC-SIGN was expressed in all DC subsets but its expression was significantly higher in IL-4 DCs (Fig 2B). In contrast, Glut-1 expression was similar in all three DC subsets (Fig 2C). These results suggest that HTLV-1 binding might be lower in IFN-α DC and TGF-β compared to IL-4 DC. We therefore measured viral capture using detection of p19^gag^ in the DC populations 3h after contact with C91-PL infected cells (Fig 2D) in addition to productive infection determined 3 days post contact (Fig 2E). Surprisingly, capture was 6-fold higher in IFN-α DCs and 3 fold higher in TGF-β DCs compared to IL-4 DCs (Fig 2D). This suggests that viral capture is inversely correlated to productive infection (Fig 2E), and could be independent of NRP-1 and/or DC-SIGN expression level. Thus, IFN-α DC resistance to HTLV-1 infection is not linked to decreased capture efficiency or to a lower expression of viral fusion receptor.

### Exposure to type I IFN does not restrict infection of IL-4 DCs

**Fig 3:**
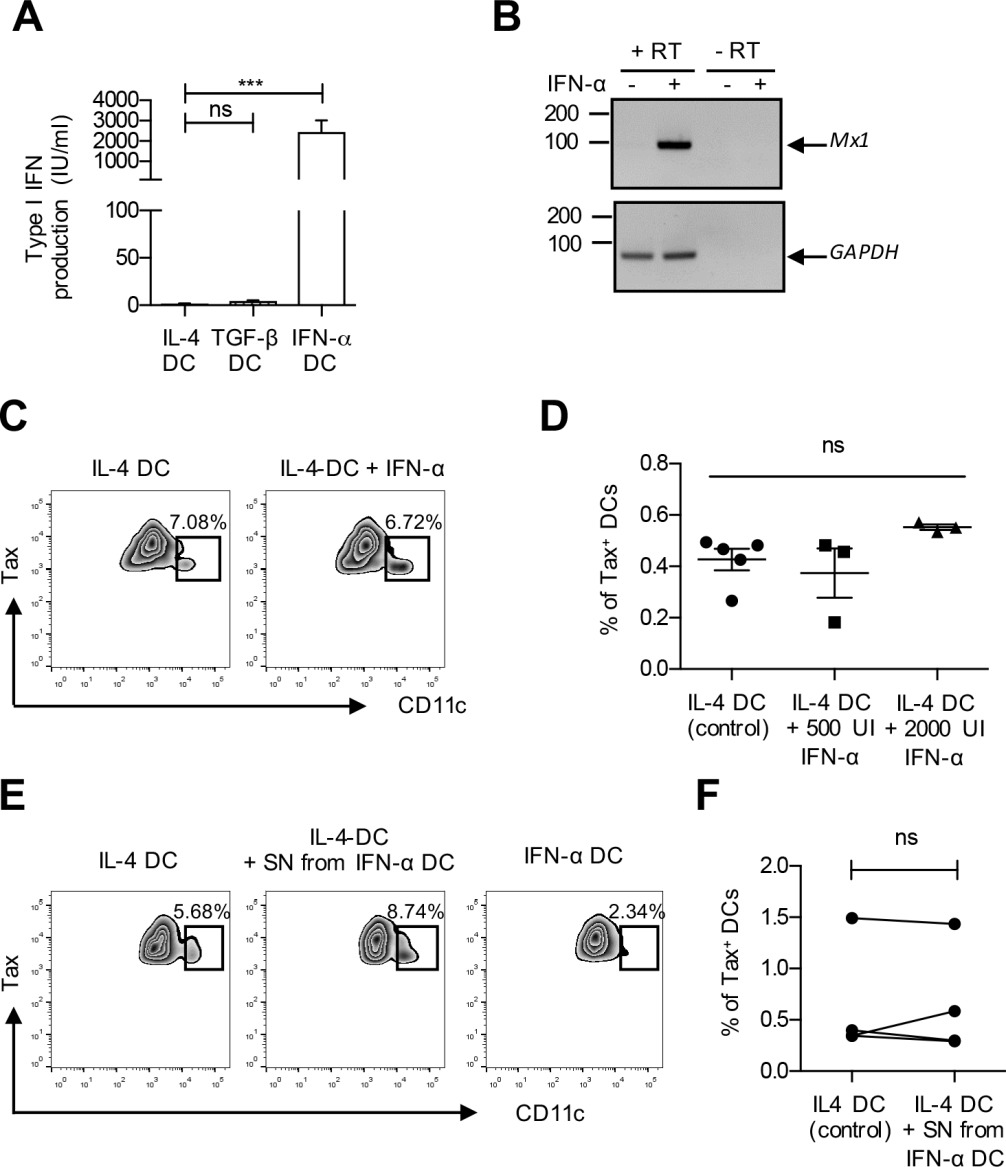
Recombinant IFN-α or supernatant from IFN-α DCs is inefficient to restrict infection of IL-4 DCs. **(A)** Supernatant from IL-4, TGF-β and IFN-α DCs derived from the same donors were collected after differentiation and type I IFN levels measured using HL116 reporter cells expressing ISRE-Luc. Asterisks indicate statistically significant differences calculated using one-way ANOVA followed by Bonferroni’s multiple comparison test: *** p<0.001; ns: non significant. **(B)** IL-4 DCs were treated with 500 IU/ml of recombinant IFN-α for 18 h, or left untreated as a control. Total RNA was extracted and retro-transcribed (RT). PCR was performed using *Mx-1* or *GADPH* specific primers. Absence of DNA contamination was controlled using PCR amplification on total RNA in the absence of RT treatment. **(C)** IL-4 DCs were treated or not with recombinant IFN-α (500 or 2000 IU/ml) before co-culture with mitomycin-treated C91-PL cells for 3 days, and Tax expression was measured. Cells from the co-culture were first gated on Tax expression and then on CD11c expression. The Tax-expressing DC population is shown in the box, as well as the percentage of Tax expressing DC in the Tax positive population. A representative experiment is shown as zebra plots. **(D)** The percentage of infected CD11c^+^ Tax^+^ DCs was determined after treatment of IL-4 DCs with increasing amounts of recombinant IFN-α 2a. Results from at least 3 independent experiments with different donors are shown. Statistical analysis was performed using one-way ANOVA: ns: non significant. **(E)** IL-4 DCs were treated or not with the supernatant from IFN-α DCs before co-culture with mitomycin-treated C91-PL cells for 3 days, and Tax expression was measured. IFN-α DCs were included as a control. Cells were then gated and results represented as in (C). **(F)** The results from 4 independent experiments obtained with different donors are presented as in (D). Statistical analysis was performed using a t-test: ns: non significant.

It is well established that type I IFN controls several viral infections through the induction of numerous interferon-inducible genes (ISGs) that restrict viral cycle at different levels [26]. Furthermore, HTLV-1 infection is restricted when target T-cells are treated with IFN-α before contact with the virus [23,27]. We therefore measured the basal level of IFN-α production by the three different DC subtypes. As expected, IFN-α DCs produced and released higher levels of type I IFN compared to IL-4 or TGF-β DCs (Fig 3A).

Given that IFN-α production by IFN-α DCs could be responsible for their resistance to HTLV-1 infection, we treated IL-4 DCs with increasing doses of recombinant IFN-α 2a before co-cultivating them with C91-PL infected cells. We first verified that recombinant IFN-α treatment did not affect IL-4 DC phenotype (S2A Fig) and was sufficient to induce expression of ISGs as measured by the up-regulation of *Mx-1* mRNA (Fig 3B).

Surprisingly however, treatment of IL-4 DCs with recombinant IFN-α did not affect their susceptibility to HTLV-1 as exemplified by the number of CD11c-positive Tax-positive cells shown for a representative experiment (Fig 3C) and the percentage of infected DCs observed in different experiments performed with cells from several independent donors (Fig 3D), or by the number of p19^gag^-positive cells after 3h of DCs exposure to C91-PL cells (S2B Fig). These results suggest that IL-4 DC exposure to recombinant IFN-α 2α does not affect their susceptibility to HTLV-1 infection.

Type I IFN includes IFN-β and 12 different IFN-α proteins that have different antiviral properties. IFN-α 2a was recently shown to have the weakest antiviral activity against HIV-1 compared to eight others [28]. Thus, recombinant IFN-α 2a used above might not be sufficient for the restriction of HTLV-1 infection in IFN-α DCs. To address this issue, we tested the ability of the physiological type I IFN-α present in the supernatant of IFN-α DCs to restrict HTLV-1 infection in IL-4 DCs. Again, the number of Tax positive IL-4 DCs did not differ significantly when cells were cultured for 18 h with or without the supernatant from IFN-α DCs (Fig 3E and 3F), confirming that type I IFN produced by IFN-α DCs is not responsible for the resistance of IFN-α DCs to HTLV-1 infection.

### Decreased susceptibility to HTLV-1 infection is linked to DC maturation

**Fig 4:**
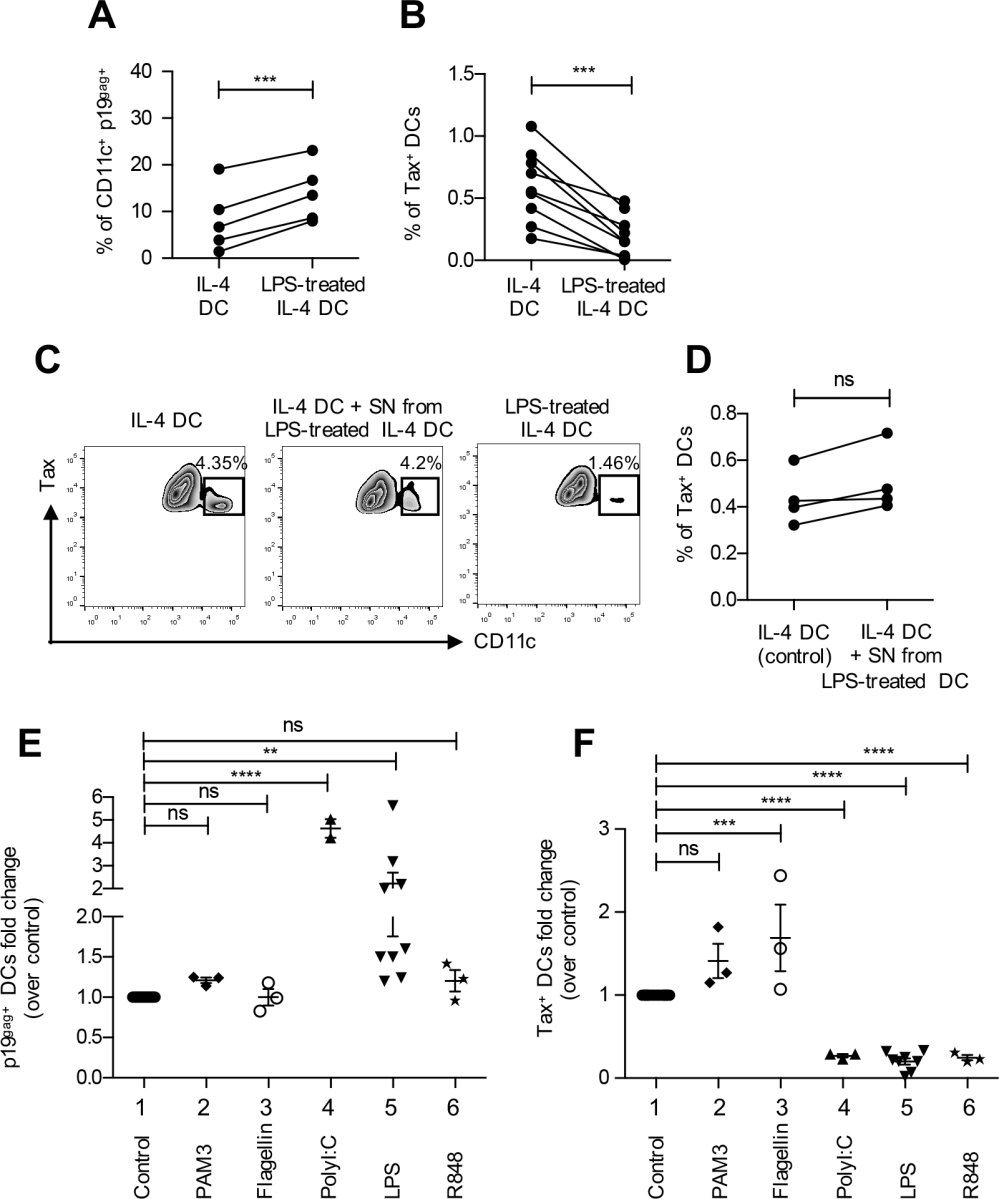
DC maturation is responsible for restriction of HTLV-1 productive infection. **(A)** IL-4 DCs were treated with the TLR-4 ligand LPS before co-culture with C91-PL for 3 h, and viral capture measured by flow cytometry using p19^gag^ detection. Untreated IL-4 DCs were used as a control. The percentage of DCs expressing p19^gag^ is shown for immature IL-4 DCs and LPS-treated IL-4 DCs from the same donor. Results from 5 independent experiments performed on different donors are shown. Statistical analysis was performed using a t-test: ***<0.001. **(B)** IL-4 DCs were treated with LPS before co-culture with mitomycin-treated C91-PL cells for 3 days, and productive infection measured by flow cytometry using Tax protein detection. Untreated IL-4 DCs were used as a control. The percentage of DCs expressing Tax is shown for immature IL-4 DCs and LPS-treated IL-4 DCs from the same donor. Paired results from 9 independent experiments performed on different donors are shown. Statistical analysis was performed using a t-test: *** p< 0.001. **(C)** IL-4 DCs were treated or not with the supernatant from LPS-treated DCs (middle plot) before co-culture with mitomycin-treated C91-PL cells for 3 days, and Tax protein expression was measured. LPS-treated DCs were included as a control (right plot). Cells from the co-culture were first gated on Tax expression and then the infected DC population expressing Tax was determined on CD11c expression. A representative experiment is shown as zebra plots with the infected Tax^+^CD11c^+^ DC population squared, and the percentage of Tax expressing DCs in the Tax positive population is indicated. **(D)** The percentage of DCs expressing Tax was measured for IL-4 DCs (control) or for IL-4 DCs treated with the supernatant from LPS-treated DCs from the same donor co-cultured with mitomycin-treated C91-PL cells for 3 days. Results from 4 independent experiments performed on different donors are shown. Statistical analysis was performed using a t-test: ns: non significant. **(E)** IL-4 DCs were left untreated as controls or treated with the TLR-2 ligand PAM3CSK4, the TLR-5 ligand flagellin, the TLR-3 ligand PolyI:C, the TLR-4 ligand LPS or the TLR-7/8 ligand R848 before co-culture with mitomycin-treated C91-PL cells for 3 h, and viral capture measured by flow cytometry using p19^gag^ staining. The percentage of DCs stained for p19^gag^ was determined and normalized to that of control IL-4 DCs. Results from at least 3 independent experiments performed on different donors are represented as fold increase over control. Asterisks indicate statistically significant differences calculated using one-way ANOVA followed by Bonferroni’s multiple comparison test: **** p< 0.0001, ** p<0.01, ns: non significant. **(F)** IL-4 DCs were left untreated or treated with the TLR-2 ligand PAM3CSK4, the TLR-5 ligand flagellin, the TLR-3 ligand PolyI:C, the TLR-4 ligand LPS or the TLR-7/8 ligand R848 before co-culture with mitomycin-treated C91-PL cells for 3 days, and productive infection measured by flow cytometry using Tax expression. The percentage of Tax expressing DCs was determined and normalized to that of control IL-4 DCs. Results from at least 3 independent experiments performed on different donors are represented as fold increase over control. Asterisks indicate statistically significant differences calculated using one-way ANOVA followed by Bonferroni’s multiple comparison test: **** p<0.0001, *** p< 0.001, ns: non significant.

IFN-α DCs are more mature than IL-4 DCs, as shown by the up-regulation of HLA-DR and CD40 markers (S1A Fig and [19]). We thus asked whether DC maturation could account for restriction of HTLV-1 infection in IFN-α DCs. To test this hypothesis, IL-4 DCs were treated with LPS, a TLR-4 agonist, which induces the up-regulation of DC maturation markers such as HLA-DR, CD40 and CD86 (S3A Fig). Both immature and LPS-matured IL-4 DCs were then exposed to C91-PL cells for 3 h to measure viral capture, or for 3 days to measure productive infection. Similar to our results with IFN-α DCs, the percentage of p19^gag^-positive DCs was significantly higher in LPS-matured DCs (Fig 4A), while the percentage of Tax positive DCs was significantly lower when cells had been matured with LPS (Fig 4B). This indicates that LPS treatment of IL-4 DCs increases HTLV-1 uptake but restricts their productive infection and seems to recapitulate IFN-α DC restriction of HTLV-1. LPS-treatment of IL-4 DCs induced their maturation and was also accompanied with type I IFN production (S4B Fig). We thus tested whether type I IFN production after LPS-treatment could account for the decreased level of HTLV-1 productive infection. IL-4 DCs were treated with LPS for 3 h. Medium was then removed, cells were extensively washed to remove any trace of LPS and cultured for 18 h in LPS-free medium. Culture supernatant was then collected and added to another set of the same autologous IL-4 DCs. Addition of this supernatant induced an increase in CD86 expression level although lower to that of fully matured DCs, without any other change in the expression of CD40 or HLA-DR maturation markers (S3A Fig). Interestingly, treatment of IL-4 DCs with the supernatant from LPS-matured DCs did neither affect DC susceptibility to HTLV-1 as shown using a result from one representative experiment (Fig 4C) nor the percentage of infected DCs observed in different experiments performed on independent donors (Fig 4D and S3B Fig), or the number of p19^gag^-positive cells (S3C Fig). These results suggest that maturation rather than type I IFN production accounts for the decreased susceptibility of LPS-matured DCs to HTLV-1 infection. To confirm this hypothesis, we induced IL-4 DC maturation with different TLR agonist treatments. Stimulation of IL-4 DCs with PolyI:C, R848 or LPS, that are agonists of TLR-3, TLR-7/8 or TLR-4 respectively, induced a fully mature phenotype, as measured by the up-regulation of HLA-DR, CD86 and CD40 (S4A Fig) and type I IFN production (S4B Fig). In contrast, stimulation of IL-4 DCs with PAM3 or flagellin that are ligands of TLR-2 or TLR-5 respectively, induced an incomplete mature phenotype (S4A Fig) with no type I IFN production (S4B Fig). Interestingly, while treatment with ligands of TLR-3 or TLR-4 significantly increased viral capture (Fig 4E lanes 4 and 5), treatment with ligands of TLR-3, TLR-7/8 or TLR-4 significantly decreased productive infection (Fig 4F, lanes 4–6). In contrast, treatment with ligands of TLR-2 or TLR-5 did not affect viral capture (Fig 4E, lanes 2–3) but led to an increased productive infection of IL-4 DCs (Fig 4F, lanes 2–3). Altogether, these results suggest that DC maturation independently of type I IFN present in the supernatant of matured DC is responsible for the restriction of HTLV-1 infection in DCs.

### HTLV-1 is stored in similar vesicles in both permissive and restricted DCs

**Fig 5:**
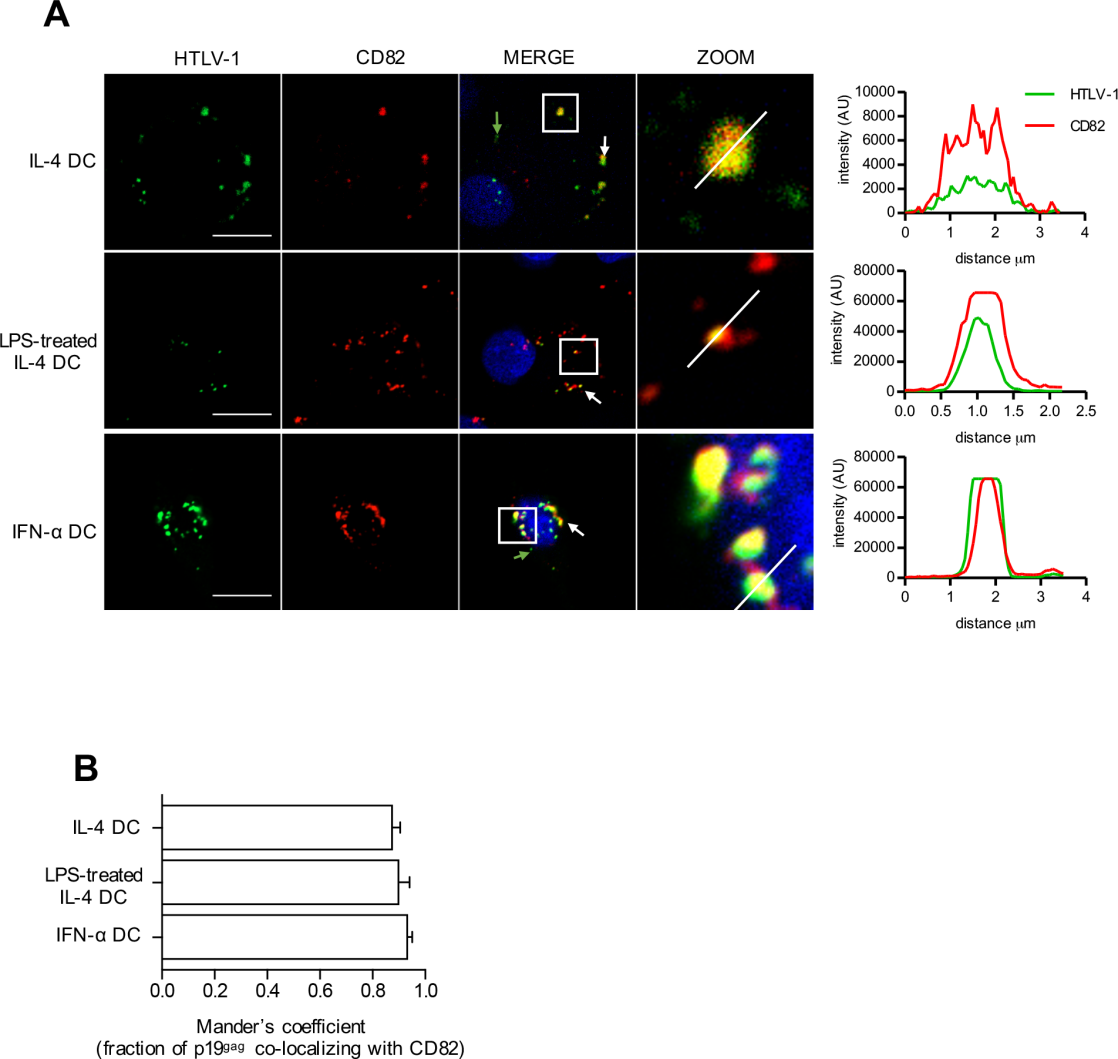
HTLV-1 localizes in CD82^+^ vesicles after capture by DCs. **(A)** IL-4 DCs, LPS-treated IL-4 DCs and IFN-α DCs were co-cultured with mitomycin-treated C91-PL cells for 3 h, and immunostained for HTLV-1 (green) and CD82 (red) before analysis by confocal microscopy. Nuclei were counterstained with DAPI (blue). White arrows show co-localization of HTLV-1 and CD82. Green arrows show vesicles stained with HTLV-1 only. Representative images obtained by confocal microscopy are shown. HTLV-1 and CD82 signals were quantified along the segment represented on the zoom panel and plotted on the histogram. Scale bars = 10 μm. **(B)** Co-localization of HTLV-1 signal with CD82 signal was calculated using Mander’s coefficient.

HTLV-1 capture is more important in mature DCs but is not linked to a productive infection. We therefore investigated whether after its capture, HTLV-1 virus might be stored in different compartments according to the different DC subsets. After 3 h of contact with infected cells, p19^gag^ signal appeared as cytoplasmic punctae in the three DC subsets (Fig 5A) suggesting a vesicular localization. None of the p19^gag^ vesicles were co-stained with the EE1A marker of early endosomes in any DC subset (S5 Fig). In contrast, most virus-containing vesicles also stained positive for the tetraspanin CD82 marker of multivesicular bodies (MVBs, Fig 5A, see white arrows), with only 10% of virus-containing vesicles that did not stain positive for CD82 (Fig 5A, see green arrows). Interestingly, the same percentage of CD82-positive virus-containing vesicles was observed in the three DC subsets (Fig 5B), suggesting that HTLV-1 might be stored in similar compartments despite a different infection outcome.

### HTLV-1 uses partially overlapping entry routes in IL-4 and IFN-α DCs

**Fig 6:**
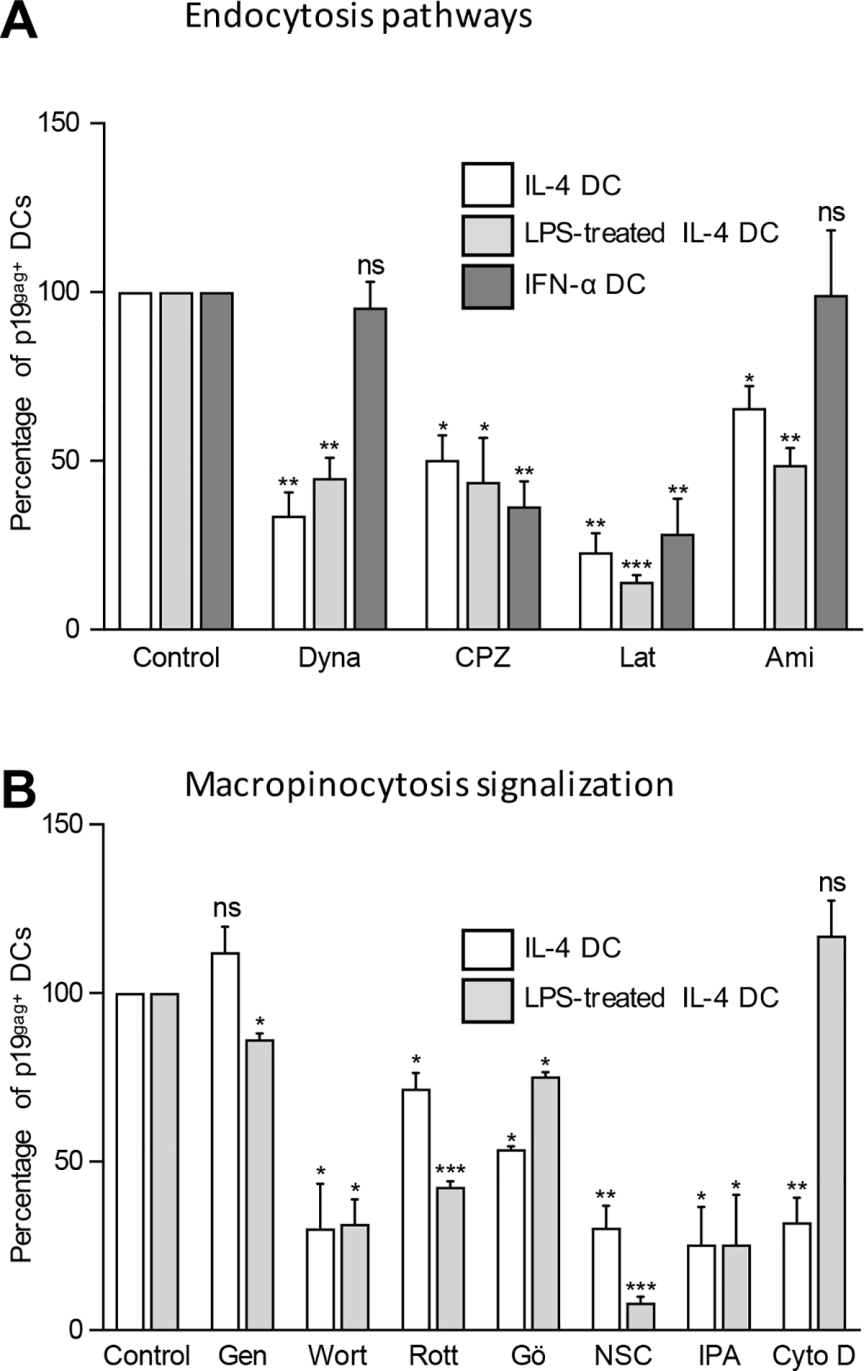
HTLV-1 uses macropinocytosis pathways to enter into IL-4 DCs and LPS-stimulated IL-4 DCs, but not in IFN-α DCs. **(A-B)** IL-4 DCs (white bars), LPS treated IL-4 DCs (light gray bars) or IFN-α DCs (dark gray bars) were treated for 3 h with **(A)** clathrin-mediated endocytosis inhibitors (chloropromazine, CPZ), dynamin II inhibitors (dynasore, Dyna), actin polymerization inhibitors (latrunculin, Lat) or macropinocytosis inhibitors (amiloride, ami) or **(B)** constitutive macropinocytosis inhibitors (genistein, Gen), PI3K inhibitor (wortmanin, Wort), PKC inhibitors (rottlerin, Rott and Gö6976), Rac-1 inhibitors (NSC23766, NSC), Pak-1 inhibitor (IPA-3, IPA), and actin inhibitor (Cytochalasin D, Cyto D). After treatment, DCs were co-cultured with mitomycin-treated C91-PL cells for 3 h and viral capture determined by flow cytometry using p19^gag^ staining. DCs treated with DMSO, ethanol or medium depending on the solvent used for inhibitor solubilization (see material and methods section) were used as controls. The percentage of DCs positive for p19^gag^ was determined for each condition and normalized to that of the untreated control DCs. Results were obtained from 3 independent experiments performed with different blood donors. Asterisks indicate statistically significant differences between treated and untreated DCs calculated using a t-test: * p<0.05; ** p<0.01;*** p<0.001; ns: non significant.

To explain the differential susceptibility to HTLV-1 infection, we hypothesized that viral entry mechanisms might differ in IL-4 DCs vs. LPS-matured and IFN-α DCs. Vesicular entry pathways are mediated by several mechanisms of endocytosis that can be blocked by pharmacological inhibitors. Hence, immature IL-4 DCs, LPS-matured IL-4 DCs or IFN-α DCs were treated with inhibitors targeting dynamin-mediated and clathrin-mediated endocytosis (dynasore and chlorpromazine respectively), caveolin-mediated endocytosis (methyl-β-cyclodextrin and nystatin), actin polymerization (latrunculin, cytochalasin D) or macropinocytosis (amiloride) before being co-cultured with C91-PL infected cells. We controlled that treatment with inhibitors did not change the viability of the different DC subsets compared to untreated DCs (S6A and S6B Fig). Viral capture was measured after 3 h of contact with C91-PL. Treatment with methyl-β-cyclodextrin and nystatin, that depleted cholesterol as controlled by the lack of lipids droplets stained by Nile red in IL-4 treated DCs (S6C Fig), did not affect p19^gag^ levels (S6D Fig), suggesting that HTLV-1 entry does not follow caveolin-mediated endocytosis. However, HTLV-1 entry was significantly reduced by both chlorpromazine and latrunculin (Fig 6A), suggesting a requirement for clathrin-mediated endocytosis and actin polymerization. Interestingly, HTLV-1 entry was significantly reduced by amiloride and dynasore in immature IL4 DCs and LPS-matured DCs but not in IFN-α DCs (Fig 6A), suggesting the requirement of macropinocytosis specifically in immature and mature IL-4 DCs. Macropinocytosis involves PI3K and PKC that control actin polymerization through the activation of Rac 1 GTPase. Rac 1 GTPase in turn controls the polymerization of actin through the activation of Pak-1. All these processes finally lead to the extension of filopodia that fold back onto the cell membrane to form large irregular macropinosomes [29]. HTLV-1 entry was blocked in immature and LPS-matured IL-4 DCs by the PI3K inhibitor wortmanin, the PKC inhibitors rottlerin and Gö6976, and inhibitors of Rac 1 (NSC23766) and Pak1 (IPA-3) (Fig 6B). This further confirms the implication of macropinocytosis in HTLV-1 entry in these subsets. Interestingly, compared to entry in IL-4 DCs, HTLV-1 entry in LPS-matured DCs was more reduced after treatment with genistein, rottlerin and NSC, less reduced after treatment with Gö6976 and not blocked by the inhibitor of actin fibers elongation cytochalasin D (Fig 6B), suggesting the involvement of a different macropinocytosis process in LPS-matured DCs. Finally, HTLV-1 entry in immature and LPS-matured IL-4 DCs was not blocked by nocodazole and blebstatin, suggesting that microtubule or myosin contraction are not involved in the macropinocytosis process of HTLV-1 entry (S6E Fig). Altogether, these results demonstrate that HTLV-1 entry in immature and LPS-matured IL4-DCs requires macropinocytosis as well as clathrin-mediated endocytosis while entry in IFN-α DC used mainly clathrin-mediated endocytosis.

### Acidic pH of vesicles decreases DC infection

**Fig 7:**
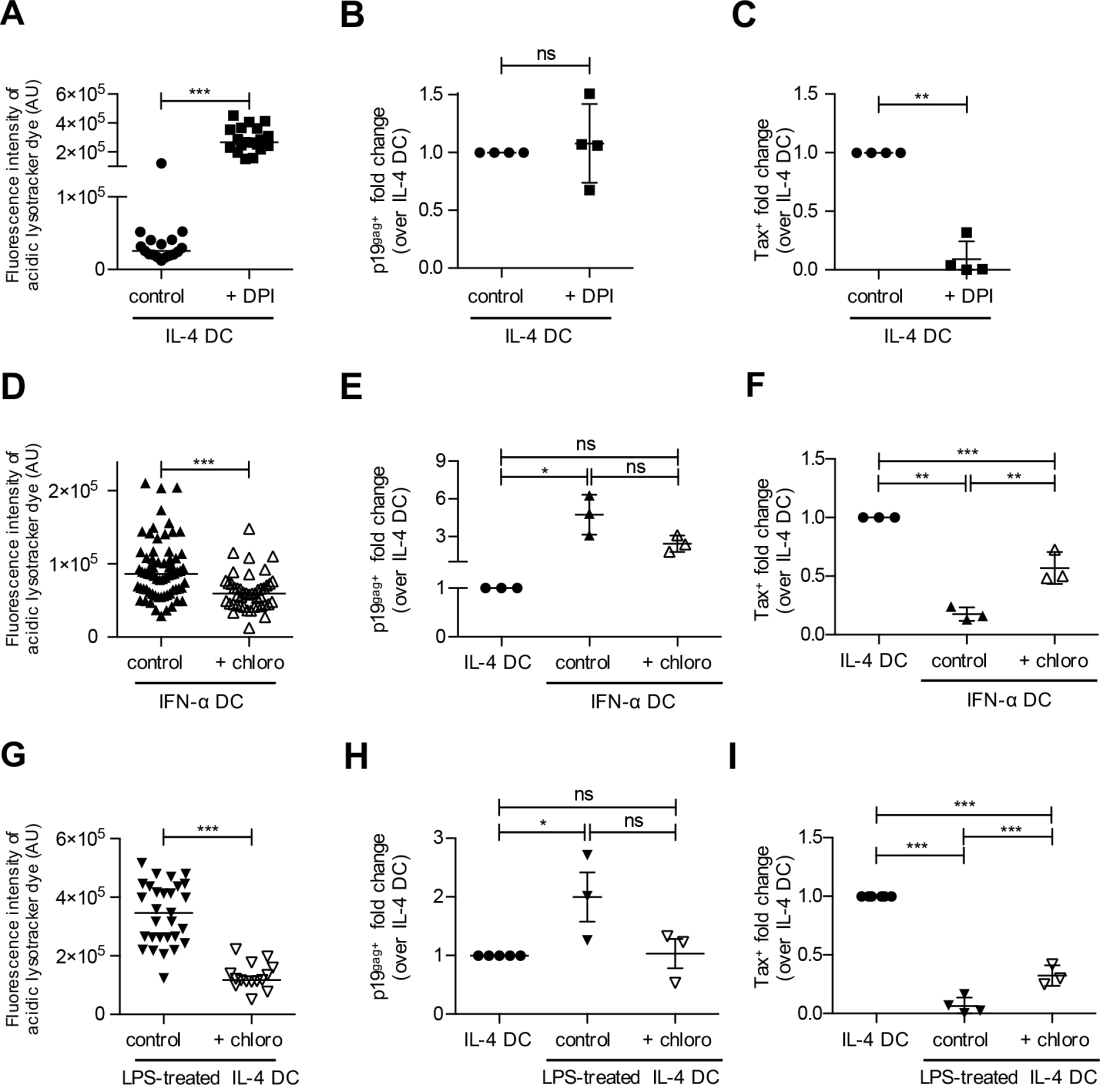
Acidic pH of vesicles decreases DC infection. **(A)** IL-4 DCs were treated with 10μM DPI for 3 h and incubated for 30 minutes with a pH-sensitive lysotracker fluorescent dye. Fluorescence intensity was determined using ImageJ. Results are representative of at least three independent experiments performed on different donors. Asterisks indicate statistically significant differences calculated using a t-test: *** p<0.001. **(B)** DPI-treated IL-4 DCs or untreated IL-4 DCs control were co-cultured with mitomycin-treated C91-PL cells for 3 h. Viral capture was measured by flow cytometry using p19^gag^ staining. The percentage of p19^gag^-positive DCs was normalized to that of control IL-4 untreated DCs and presented as fold changes over control. Results are representative of 4 independent experiments performed on different donors. Asterisks indicate statistically significant differences calculated using a t-test: ns: non significant. **(C)** DPI-treated IL-4 DCs or untreated IL-4 DCs control were co-cultured with mitomycin-treated C91PL cells for 3 days. Productive infection was measured by flow cytometry using Tax staining. The number of Tax-expressing DCs was normalized to that of control IL-4 untreated DCs and presented as fold changes over control. Results are representative of 4 independent experiments performed on different donors. Asterisks indicate statistically significant differences calculated using a t-test: ** p<0.01. **(D)** IFN-α DCs were treated for 3 h with 150 μM chloroquine, and incubated with lysotracker fluorescent dye for 30 minutes. Fluorescence intensity determined using ImageJ was compared using a t-test: *** p<0.001. Results are representative of at least 3 independent experiments performed on different donors. **(E)** Chloroquine-treated IFN-α DCs or untreated IL-4 and IFN-α DCs used as controls were co-cultured with C91-PL for 3 h. Viral capture was measured by flow cytometry using p19^gag^ staining. The percentage of p19^gag^-positive DCs was normalized to that of control IL-4 untreated DCs and presented as fold change over control. Results are representative of at least 3 independent experiments performed on different donors. Asterisks indicate statistically significant differences calculated using one-way ANOVA followed by Bonferroni’s multiple comparison test: * p<0.05 ns: non significant. **(F)** Chloroquine-treated IFN-α DCs or untreated IL-4 and IFN-α DCs used as controls were co-cultured with mitomycin-treated C91-PL cells for 3 days. Productive infection was measured by flow cytometry using Tax detection. The number of Tax-expressing DCs was normalized to that of control IL-4 untreated DCs and presented as fold changes over control. Results are representative of 3 independent experiments performed on different donors. Asterisks indicate statistically differences calculated using one-way ANOVA followed by Bonferroni’s multiple comparison test: ***p<0.001, **p<0.01. **(G)** LPS-treated IL-4 DCs were treated for 3 h with 150 μM chloroquine, and incubated with lysotracker fluorescent dye for 30 minutes. Fluorescence intensity was determined using ImageJ and compared using a t-test: *** p<0.001. Results are representative of at least 3 independent experiments performed on different donors. **(H)** Chloroquine-treated LPS-matured IL-4 DCs or untreated IL-4 and LPS-matured IL-4 DCs used as controls, were co-cultured with mitomycin-treated C91-PL cells for 3 h. Viral capture was measured and presented as in (E). Results are representative of 3 independent experiments performed on different donors. Asterisks indicate statistically differences calculated using one-way ANOVA followed by Bonferroni’s multiple comparison test: * p<0.05, ns: non significant. **(I)** Chloroquine-treated LPS-matured IL-4 DCs or untreated IL-4 and LPS-matured IL-4 DCs used as controls, were co-cultured with mitomycin-treated C91-PL cells for 3 days. Productive infection was measured and presented as in (F). Results are representative of 4 independent experiments performed on different donors. Asterisks indicate statistically significant differences calculated using one-way ANOVA followed by Bonferroni’s multiple comparison test: ***p<0.001.

Since HTLV-1 entry pathways, and intracellular localization are similar in immature IL-4 DCs that are susceptible to infection and in IFN-α DCs that are resistant to infection, we next wondered whether intravesicular pH could modify the ability of the virus to establish a productive infection. Indeed, previous studies reported that immature DCs have vesicles with neutral pH, while vesicles pH is more acidic in mature DCs [30]. Using acidic lysotracker, which fluorescence intensity is dependent on acidic pH, and using drugs that induce intravesicular acidification (diphenylene-iodonium DPI) or alkalization (chloroquine), we were able to modulate and monitor the pH of vesicles. The neutral pH of vesicles in immature IL-4 DC was acidified after DPI treatment (Fig 7A), while the slightly more acidic pH of vesicles in IFN-α DCs was alkalized after chloroquine treatment (Fig 7D). As a control, the acidic pH of vesicles in LPS-treated DCs was alkalized after choloroquine treatment (Fig 7G). IL-4 DCs treated with DPI captured similar amount of HTLV-1 than untreated IL-4 DCs when co-cultured with C91PL (Fig 7B). However, productive infection was significantly reduced under these conditions (Fig 7C). This suggests that HTLV-1 trafficking into acidic vesicles inhibits productive infection of IL-4 DCs. In contrast, in the same co-culture conditions, chloroquine treatment of IFN-α DCs, reduced viral capture to a level similar to that of IL-4 DCs, (Fig 7E). In addition, Tax expression was significantly increased in chloroquine-treated IFN-α DCs compared to untreated IFN-α DCs, although it did not reach the level of Tax expression in IL-4 DCs (Fig 7F). This suggests that productive infection is only partially restored by alkalization of IFN-α DC vesicles. Similar results were obtained after treatment of LPS-matured DCs before their co-culture with C91-PL (Fig 7H and 7I), further suggesting that alkalization of matured DC vesicles partially restores productive infection. Altogether, our results suggest that productive infection of DCs by HTLV-1 requires a neutral pH of vesicles and an immature phenotype of the DCs.

### Productive infection of DCs is required for viral transfer to T-cells

**Fig 8:**
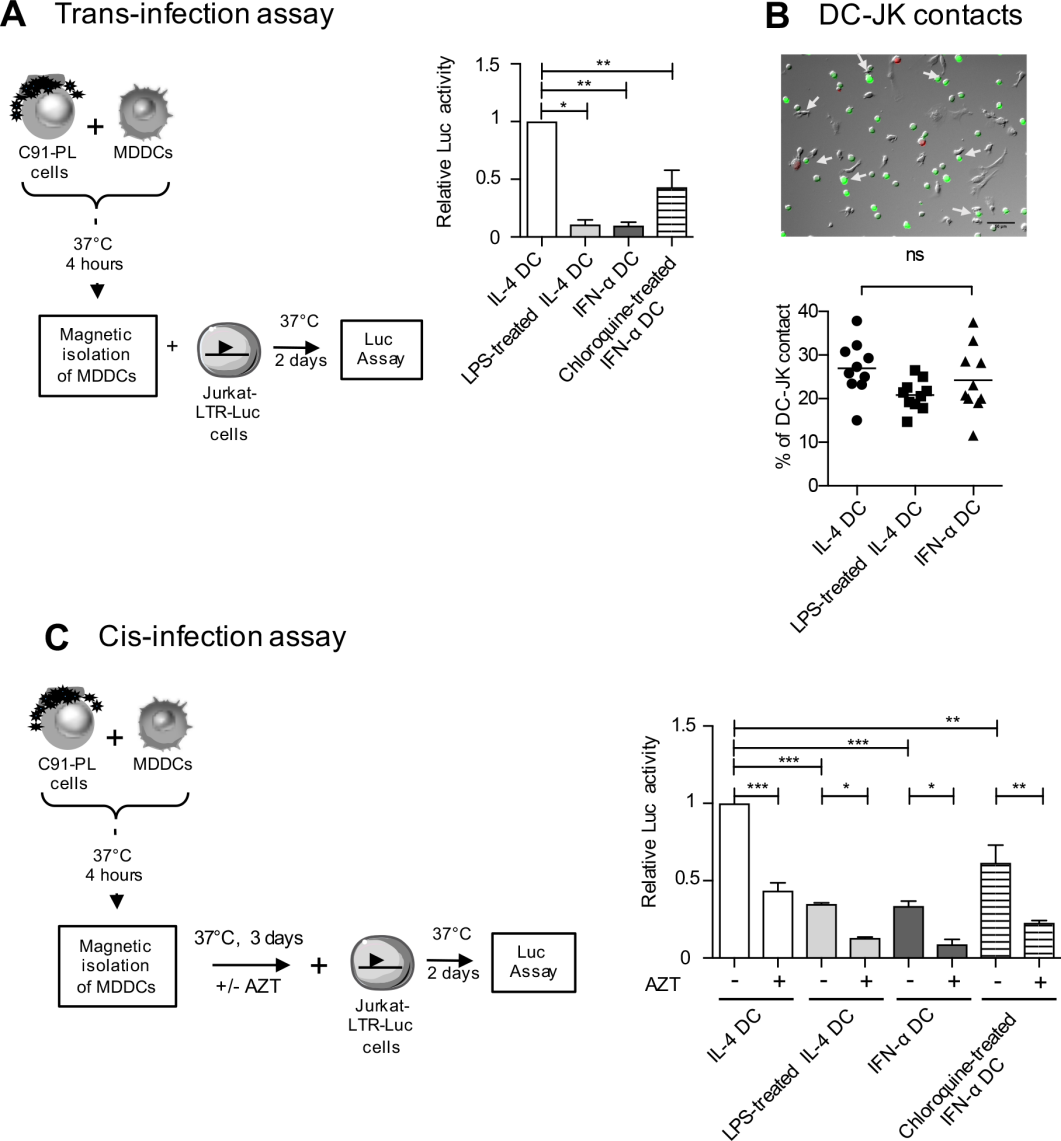
HTLV-1 is transferred to reporter T-cells from IL-4 DCs but not from LPS-treated or IFN-α DCs. **(A)** DCs were exposed to mitomycin-treated C91-PL cells for 4 h and isolated from C91-PL using positive selection with magnetic beads coated with anti BDCA-4 antibodies. Then, purified MDDCs were co-cultured with Jurkat-LTR-Luc cells and luciferase activity was assessed after 2 days. When indicated, IFN-α DCs were treated with chloroquine before their exposure to C91-PL. The graph shows the means and the standard deviations from 3 independent experiments obtained with different donors. Background signal due to leaky luciferase activity of Jurkat-LTR-Luc cells was subtracted and results were normalized to the luciferase activity of Jurkat-LTR-Luc measured after co-culture with IL-4 DCs and presented as fold change over control. Asterisks indicate statistically significant differences calculated using one-way ANOVA followed by Bonferroni’s multiple comparison test: * p<0.05, ** p<0.01. **(B)** DCs were mixed with GFP-transduced Jurkat-LTR-Luc cells and C91-PL cells stained with a red cell tracker, and contacts were visualized by fluorescence microscopy. Arrows indicate contacts between unstained DCs and GFP-transduced Jurkat-LTR-Luc cells (JK). Scale bar = 50μm. Contacts between DCs and GFP-transduced Jurkat-LTR-Luc cells were manually counted, normalized to the number of DCs in the picture analyzed, and represented as percentages. Results are representative of three independent experiments performed on different donors. Statistics were performed using one-way ANOVA. ns: non significant. **(C)** DCs were exposed to mitomycin-treated C91-PL cells for 4 h, isolated by positive selection and cultured for 3 days, in presence or absence of AZT. Then, Jurkat-LTR-Luc cells were added and luciferase activity was assessed 2 days later. When indicated, IFN-α DCs were treated with chloroquine before their exposure to C91-PL. The graph shows the means and the standard deviations from 3 independent experiments obtained with different donors. Results were normalized to the luciferase activity of Jurkat-LTR-Luc measured after co-culture with IL-4 DCs and presented as fold changes over control. Asterisks indicate statistically significant differences calculated using one-way ANOVA followed by Bonferroni’s multiple comparison test: ***p<0.001, ** p<0.01, *p<0.05.

We have previously shown that productive infection of IL-4 DCs was the main route of HTLV-1 transmission to lymphocytes, although passive transfer from HTLV-1-exposed IL-4 DCs in presence of AZT may have occurred in rare cases [9]. This passive transfer, also known as “trans-infection” (for a review see [31]), results from transmission of captured viral particles without productive infection, and is the main route used by HIV-1 for spreading to T-cells [32]. Since viral capture is around three-fold higher in LPS-matured DCs (see Fig 4E) and six-fold higher in IFN-α DCs (see Fig 2D) than in IL-4 DCs, we hypothesized that trans-infection might occur preferentially with mature DCs compared to IL-4 DCs. IL-4 DCs, LPS-treated DCs or IFN-α DCs were thus exposed to C91-PL for 3 h to allow viral capture, and then separated from C91-PL using positive selection with anti-BDCA-4 antibodies. We controlled that the DC phenotype was not changed after the purification process (S7A and S7B Fig) and allowed the removal of at least 95% of C91-PL (S7C Fig). Purified DCs were then co-cultured for 2 days with Jurkat LTR-Luc reporter cells, (Fig 8A). Luciferase signals significantly decreased when co-culture was performed with LPS-treated IL-4 DCs or IFN-α DCs, compared to co-culture with IL-4 DCs (Fig 8A). Interestingly, when IFN-α DCs were treated with chloroquine before co-culture with C91-PL, luciferase signals were significantly higher compared to co-culture with untreated IFN-α DCs (Fig 8A see graph). These results suggest that although HTLV-1 capture is higher in mature DCs, internalized virus in acidic vesicles cannot be transferred to T-cells. To control that the decreased luciferase signals were not due to a decreased ability of IFN-α or LPS-treated DCs to engage cell-to-cell contacts with Jurkat-LTR-Luc cells, we counted the number of DC-Jurkat-LTR-Luc conjugates in co-cultures containing DCs, C91-PL and Jurkat-LTR-Luc cells. To distinguish the different cell types in the co-culture, C91-PL were stained with a cell tracker and appeared in red, while Jurkat-LTR-Luc cells were transduced with GFP-expressing lentivectors, and appeared in green. DCs were left unstained (Fig 8B). The number of cell conjugates between unstained DCs and GFP-labelled Jurkat-LTR-Luc cells after 3 h of co-culture did not change significantly when IL-4, IFN-α or LPS-treated DCs were used (Fig 8B, see graph), suggesting that decreased viral transfer from IFN-α DCs and LPS-treated DCs was not due to a lack of contacts with Jurkat cells. Next, IL-4 DCs, LPS-treated DCs or IFN-α DCs were exposed to C91-PL for 3 h, purified using positive selection with anti-BDCA-4 antibodies and cultured for three days to allow productive infection. Then, Jurkat-LTR-Luc reporter cells were added in the MDDCs culture and luciferase signals monitored two days later (Fig 8C). In that setting, IL-4 DCs were able to transfer significant amounts of HTLV-1 (Fig 8C), while reduced luciferase signals were detected using LPS-treated IL-4 DCs or IFN-α DCs (Fig 8C), confirming that productive infection is required to allow virus transfer to T-cells. This was further controlled using AZT treatment, which significantly reduced luciferase signals.

Surprisingly, AZT treatment of LPS-treated IL-4 DCs or IFN-α DCs also reduced luciferase signals observed with the untreated DC subsets, suggesting that the viral transfer observed with these subsets relies on their productive infection and not on long term viral capture (Fig 8C). In addition, when IFN-α DCs were treated with chloroquine before their exposure to C91-PL and their purification, viral transfer to T-cells was partially restored, but only in the absence of AZT. Altogether, these results suggest that viral transmission from DCs to T-cells mainly results from transmission of newly synthesized virus from productively infected DCs, and not from passive transmission after viral capture by LPS-matured or IFN-α DCs.

## Discussion

Apart from circulating CD4^+^ T lymphocytes that represent the main infected cell population in chronically infected individuals, HTLV-1 proviral DNA is also detected in antigen presenting cells (APCs) such as myeloid DCs [33], monocytes and plasmacytoid DCs [11]. We previously showed that *in vitro* MDDCs are more susceptible to HTLV-1 infection than autologous primary T-cells [9], supporting a model in which DCs would be the first cells to be infected during primary infection. *In vivo*, DCs come with different flavors and functions depending on their location in the body. Thus, virus interaction with DCs may have different outcomes depending on the nature of the DCs encountered in the course of infection. In this study, we used dendritic cells differentiated *in vitro* from human monocytes to generate different DC subsets mimicking those present *in vivo*. We demonstrate for the first time that DCs are not all equally susceptible to HTLV-1 infection. While immature DCs are more susceptible than tolerogenic-TGF-β DCs to HTLV-1 infection, inflammatory DCs and mature DCs are resistant to HTLV-1 infection.

Surprisingly, resistance is neither due to the presence of high level of type-I interferon produced by inflammatory DCs or mature DCs, nor to the exogenous recombinant interferon used to generate those DCs. Interestingly, these results are different from previous reports, including ours, obtained in T-cells, which showed that their susceptibility to HTLV-1 infection is sensitive to IFN-α [23,27]. Of note, cell-type-dependent susceptibility to IFN has already been reported and was linked to the use of cell-type-specific patterns of STAT activation as well as to usage of additional signaling components that can finely tune ISGs expression in a given cell type [34,35]. In addition, substantial quantitative differences in STAT activation in different cell-types may be linked to the induction of the same ISGs but with different amplitude [36]. In T-cells, treatment with exogenous IFN-α2 results in a block in HTLV-1 protein expression, due to protein kinase R (PKR) activation while the early steps of the viral cycle are maintained [23]. Strikingly, it was shown that treatment with recombinant IFN-α2 induced a higher expression of PKR in DCs than in T-cells [36]. Thus, our results, which demonstrate a lack of antiviral control in DCs after IFN treatment, suggest that PKR induction in DCs might not be as efficient as in T-cells to restrict HTLV-1 infection. Alternatively, PKR phosphorylation that is required for its antiviral activity might not be present or sufficient in DCs treated with type I IFN. In addition, PKR antiviral activity could be counterbalanced by the concomitant induction of ADAR-1 (Adenosine Deaminase Acting on RNA type 1), another ISG that has been shown to enhance replication of several viruses [37]. Interestingly, ADAR-1 expression in T-cells suppresses IFN-α inhibitory effect on HTLV-1 expression through the repression of PKR phosphorylation [38]. Thus, the inability of IFN-α to restrict HTLV-1 infection in DCs might be the result of an unbalanced expression of ADAR-1 over that of PKR.

Our results also demonstrated that restriction was not due to a lack of viral entry in mature DCs, although the level of viral binding receptors is lower than in susceptible DCs. This suggests that viral capture by DCs that have been exposed to infected cells may use other receptors. Interestingly, HTLV-1 was observed in CD82 positive compartment in all DC subsets. CD82 is a tetraspanin receptor located in MVBs and at the plasma membrane, and has been shown to interact with the HTLV-1 envelope glycoprotein in T-cells [39]. Thus, one could hypothesize that CD82 is used by HTLV-1 to enter DCs during cell-to-cell transfer, and maybe involved in the targeting of HTLV-1 to vesicles in DCs. This is currently under investigation in our laboratory. Indeed, localization of incoming viruses in vesicles in both susceptible and resistant DCs could reflect a distinct entry route in these cell types compared to T-cells, in which it is assumed that HTLV-1 entry occurs after fusion at the plasma membrane [40]. We observed that the level of the fusion receptor Glut-1 is similar in the three DC subsets, suggesting that HTLV-1 capsids could be delivered to the cytosol after fusion of the viral envelope with the membrane of the vesicles. Accordingly, both in susceptible DCs and in restricted DCs in which vesicles had been alkalized after treatment with chloroquine, Tax expression was detected. This indicates that the viral cycle can be completed after the virus had trafficked through the endocytosis pathway. In contrast, Tax was not detected in susceptible DCs in which vesicles had been acidified after treatment with DPI, thus in which incoming vesicular viruses had been inactivated. This suggests that viral fusion at the plasma membrane, even if it is present, does not allow a productive infection. Vesicular uptake of viruses may not be limited to DCs and could be a feature of cell-to-cell transmission. Indeed, such a mechanism was also reported during HIV-1 cell-to-cell transfer between T-cells [41,42,43]. In that case, viral fusion occurred in the vesicles of the target cells after the late maturation of Gag proteins, authorizing the interaction of HIV-1 envelope with its co-receptor and thus the release of HIV-1 capsids into the cytoplasm, and subsequent productive infection [44]. It is thus possible that similar entry pathways occur during HTLV-1 cell-to-cell transmission.

We showed here that maturation of DCs strongly limited their productive infection. Interestingly, it was shown that stimulation of primary hepatocytes with TLR agonists was able to inhibit replication of woodchuck hepatitis B virus (WHBV) in an interferon-independent pathway [45]. In this study, restriction was not due to the expression of a soluble factor nor to activation of JAK/STAT pathway that is specific to IFN signaling, but was induced by TLR signaling via activation of the MAPK/ERK and PI-3K/Akt pathways. It was therefore suggested that TLR-induced proteins could act as restriction factors. We cannot exclude that maturation of DCs may induce HTLV-1 specific restriction factors. However, our results suggest that the first mechanism of HTLV-1 replication restriction relies on a post entry blockade and on specific viral trafficking.

DC maturation is a complex process that modifies DC function such as antigen capture, antigen processing and presentation. Pathogen capture mainly occurs via macropinocytosis, a non-receptor mediated endocytic pathway. Macropinocytosis is a constitutive process in immature DCs [46], while it is switched to an induced process in mature DCs [47]. Indeed, in the absence of pathogen-induced signaling, mature DCs have lower abilities to internalize pathogens compared to immature DCs. Interestingly, we showed here that HTLV-1 entry was blocked by genistein, an inhibitor of induced macropinocytosis but not by cytochalasin D, a drug acting on actin fibers elongation in mature but not in immature DCs. This suggests distinct actin requirements that may be relevant to mechanisms specific to constitutive versus induced macropinocytosis.

These entry mechanisms may be consistent with the intracellular localization of HTLV-1 in the different subsets. Interestingly, earlier studies using electron microscopy analyses of MDDCs co-cultured with HTLV-1-infected lymphocytes, showed viral particles internalized in vesicles [6], although the nature of these vesicles was not identified. Our results showed that in all DCs subsets, viruses were found in large vesicles also positive for tetraspanin but not for early endosome marker. This staining is evocative of MVB, and is reminiscent of the Virus-Containing Compartments (VCC) described in HIV-1 infected macrophages [48,49,50,51,52,53,54,55,56].

**Fig 9:**
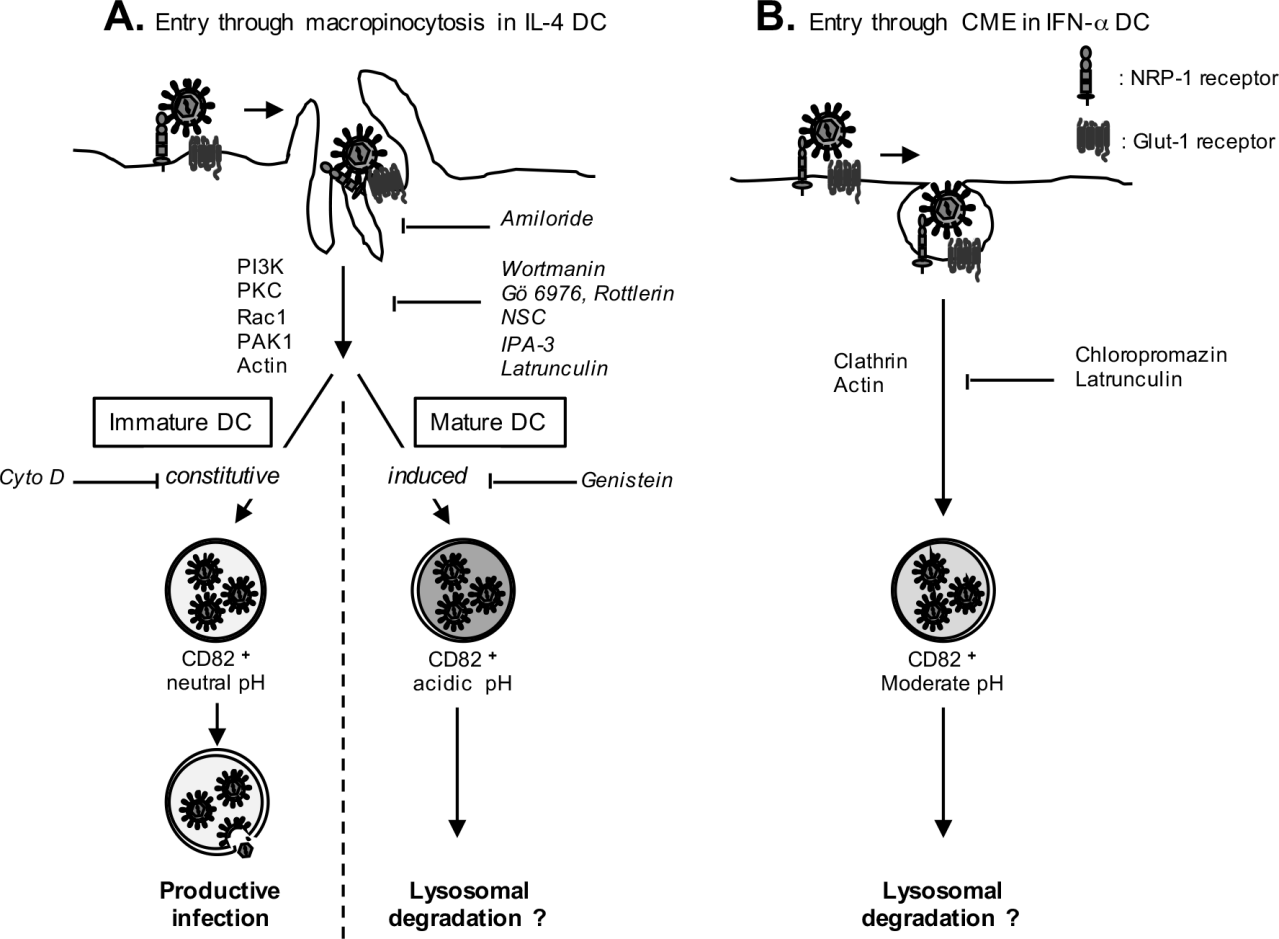
Schematic representation of HTLV-1 entry in IL-4, LPS-treated IL-4, and IFN-α DCs. **(A)** Entry through macropinocytosis in IL-4 and LPS-treated DCs. After binding to its receptors NRP-1 and Glut-1, HTLV-1 enters using the transduction pathway characteristic of macropinocytosis, that is common in IL-4 and LPS-treated DCs. Specific drugs are shown on the right side of the first arrow. Neutral pH of macropinosomes from immature DCs is indicated in light grey, while acidic pH from mature DCs is indicated in dark grey. The presence of CD82 is indicated below each macropinosome. **(B)** Entry through clathrin-mediated endocytosis (CME) in IFN-α DCs. After binding to NRP-1 and Glut-1, HTLV-1 enters using clathrin and actin pathway. Specific drugs are shown on the right side of the arrow. The pH of vesicles is indicated by the grey color. See text for details.

Thus, based on all our results, we propose a schematic model for HTLV-1 infection of DC subsets (Fig 9). When HTLV-1 encounters immature DCs, viral entry through constitutive macropinocytosis would lead to internalization of HTLV-1 in MVB-like compartments from which the neutral pH would authorize viral capsid exit and completion of the viral replication cycle (Fig 9A, left panel). In contrast, in IFN-α DCs, entry through clathrin-mediated endocytosis and not through macropinocytosis would lead to trafficking in vesicles targeted to degradation (Fig 9B). Finally, if DCs are matured before their interaction with HTLV-1, entry using constitutive macropinocytosis would deliver viruses to vesicles with an acidic pH, that would impair HTLV-1 infection (Fig 9A, right panel). Surprisingly, alkalization of vesicles from mature DCs using chloroquine restored HTLV-1 infection, but only partially with a percentage of infection around 60% lower than that observed in immature DCs. This suggests that in addition to the traffic in acidic compartments, induction of HTLV-1-specific restriction factors induced after DC maturation would insure an efficient blockade of HTLV-1 replication in mature DCs.

Are these different outcomes of HTLV-1 infection in the different DC subsets important for the virus transfer to T-cells? In the HIV-1 model, DCs are resistant to HIV-1 infection due to the presence of several restriction factors [57], and thus most of the viral transfer from DCs to T-cells occurs by trans-infection, which does not require productive infection [58]. More importantly, DC-mediated trans-infection is more efficient when HIV-1 virions have been captured by mature DCs [31,59]. Interestingly, DC maturation with TLR agonists also renders them susceptible to X4-tropic HIV-1 infection, although they are still resistant to infection with R5-tropic HIV-1 [60]. This suggests that DC infection with HIV-1 is controlled by several factors that depend upon the DC maturation status and upon HIV-1 strains. Depending on the viral strain, transfer of HIV-1 from DCs to T-cells would use either trans-infection in the case of R5-tropic viruses or cis-infection in the case of X4-tropic viruses. In any case, mature DCs would be responsible for the efficient transfer to T-cells and rapid spreading of HIV-1 within individuals [32]. During HTLV-1 infection, interaction with DCs is also important for the establishment of the chronic infection of T-cells [15,61]. However, since this infection has not been linked to immune activation, HTLV-1 might more likely interact with immature DCs. Thus, our results showing that immature DC are the only subset able to transfer the virus to T-cells, in a process mainly based on their productive infection, is relevant to the *in vivo* situation. The nature of the DCs encountered by HTLV-1 upon primo-infection, which display a differential susceptibility to HTLV-1 infection, would therefore determine the efficiency of viral transmission to T-cells. Finally, in contrast to the dissemination of HIV-1 by mature DCs, our results strongly suggest that the restriction of HTLV-1 infection in mature DCs and more importantly, their inability to transfer the virus to T-cells might be an efficient way for the host to limit HTLV-1 spread.

## Supporting information

S1 Fig Surface markers in IL-4 DCs, TGF-β DCs and IFN-α DCs. **(A)** Cells were labeled with fluorochrome-coupled antibodies (black lines) directed against CD11c, HLA-DR, CD83 and CD40. Unstained cells were used as negative controls (black graphs). **(B)** IL-4 DCs infection was determined by the number of Tax-expressing cells among IL-4 DCs in presence or absence of AZT. Results from 3 independent experiments obtained from different donors are presented. Asterisks indicate statistically significant differences calculated using a paired t-test: *p<0.05.
(TIF)

S2 Fig Recombinant IFN-α does not induce maturation of IL-4 DC and does not affect viral capture. **(A)** IL-4 DCs were cultured for 18 h in the presence of exogenous IFN-α (2000 IU/ml). Untreated (white histogram) and IFN-α treated IL-4 DCs (light grey histogram) were stained with antibodies directed against CD11c, DC-SIGN, NRP-1, HLA-DR, CD86 and CD40. Unstained cells were used as negative controls (black graphs). **(B)** Treated and untreated IL-4 DC from the same blood donor were co-cultured with mitomycin-treated C91-PL cells for 3 h and the percentage of p19^gag^-positive DCs was assessed by flow cytometry. Results are representative of 3 independent experiments performed on different donors. T-test was performed to assess statistically significant differences: ns = non significant.
(TIF)

S3 Fig Treatment of IL-4 DCs with supernatant from LPS-matured DCs does not change IL-4 DC phenotype. **(A)** IL-4 DCs from same donor were cultured for 18 h with LPS (black dotted line) or with supernatant from LPS-matured DCs (light grey histogram) or left untreated as a control (black line). Cells were labeled with antibodies against CD11c, DC-SIGN, NRP-1, HLA-DR, CD86 and CD40. Unstained cells were used as negative controls (dark grey histogram). **(B and C)** IL-4 DCs, IL-4 DCs stimulated with supernatant from LPS-treated IL-4 DCs and LPS-treated IL-4 DCs from the same donor were co-cultured with mitomycin-treated C91-PL cells for 3 h **(C)** or 3 days **(B)** and the number of Tax-positive DCs **(B)** or the percentage of p19^gag^-positive DCs **(C)** were assessed by flow cytometry. DCs were discriminated from the C91-PL population by gating on CD11c expression. The results are shown as number of Tax-positive DCs **(B)** or as percentage of p19^gag^-positive DCs **(C)**.
(TIF)

S4 Fig Phenotype of IL-4 DCs treated with TLR agonists. **(A)** IL-4 DCs were left untreated (white histogram) or were treated for 18 h with the TLR-3 ligand PolyI:C, the TLR-4 ligand LPS, the TLR-7/8 ligand R848, the TLR-2 ligand PAM3CSK4 or the TLR-5 ligand flagellin (light grey histogram). Cells were labeled with antibodies against HLA-DR, CD86 and CD40. Unstained cells were used as negative control (dark grey histogram). **(B)** Supernatant from untreated IL-4 DC or DC treated with the TLR agonists were collected after 18 h of culture and type I IFN levels measured using HL116 reporter cells expressing ISRE-Luc.
(TIF)

S5 Fig HTLV-1 is not localized in EE1A^+^ vesicles after capture by DCs. IL-4 DCs, LPS-treated IL-4 DCs and IFN-α DCs were co-cultured with mitomycin-treated C91-PL cells for 3 h, and immunostained for p19^Gag^ (red) and EE1A (green) before analysis by confocal microscopy. Nuclei were counterstained with DAPI (blue). Representative images obtained by confocal microscopy are shown. Scale bars = 10 μm.
(TIF)

S6 Fig Treatment with entry inhibitors does not affect cell viability. IL-4 DCs (black bars), LPS treated IL-4 DCs (light gray bars) or IFN-α DCs (white bars) were treated for 3 h with **(A)** dynamin II inhibitors (dynasore, Dyna), clathrin-mediated endocytosis inhibitors (chlorpromazine, CPZ), actin polymerization inhibitors (latrunculin, lat) or macropinocytosis inhibitors (amiloride, ami) or **(B)** with constitutive macropinocytosis inhibitors (genistein, Gen), PI3K inhibitor (wortmanin, Wort), PKC inhibitors (rottlerin, Rott and Gö6976), Rac-1 inhibitors (NSC23766, NSC), Pak-1 inhibitor (IPA-3, IPA), and actin inhibitor (Cytochalasin D, Cyto D). Cell viability was monitored by FACS using live-dead staining. **(C)** IL-4 DCs were left untreated (left panel) or treated (right panel) for 3 h with methyl B cyclodextrine (MβCD), fixed and incubated for 15 min with Nile red to stain lipid droplets (green). Nuclei were counterstained with DAPI (blue). Cells were visualized by fluorescence microscopy. **(D-E)** IL-4 DCs (black bars) or LPS treated IL-4 DCs (light gray bars) were treated for 3 h with nystatin or MβCD to deplete cholesterol **(D)** or nocodazole (Noco) or blebstatin (bleb) to block microtubule polymerization **(E)**. After treatment, DCs were co-cultured with mitomycin-treated C91-PL cells for 3 h and viral capture determined by flow cytometry using p19^gag^ staining. DCs treated with DMSO were used as controls. The percentage of DCs positive for p19^gag^ was determined for each condition and normalized to that of the untreated control DCs. Results were obtained from 3 independent experiments performed with different blood donors.
(TIF)

S7 Fig Phenotype of DCs and yield of C91-PL depletion after magnetic bead purification. **(A)** Untreated or **(B)** LPS-treated IL-4 DCs were exposed to C91-PL for 4 h, left untouched (before, white histograms) or magnetically isolated (after, light grey histograms) and labeled with antibodies against CD11c, DC-SIGN, HLA-DR, CD86 and CD40. Unstained cells were used as negative controls (dark grey histogram). **(C)** The yield of C91-PL depletion after magnetic isolation of IL-4 DCs was estimated by flow cytometry using anti-human CD11c staining. The percentage of C91-PL cells (CD11c negative population) and the percentage of DCs (CD11c positive population) are indicated on each plot before (left) and after (right) magnetic isolation of DCs.
(TIF)
