# Correction: Dendritic cell maturation, but not type I interferon exposure, restricts infection by HTLV-1, and viral transmission to T-cells

**DOI:** 10.1371/journal.ppat.1006494

**Published:** 2017-07-11

**Authors:** Gergès Rizkallah, Sandrine Alais, Nicolas Futsch, Yuetsu Tanaka, Chloé Journo, Renaud Mahieux, Hélène Dutartre

A sentence is omitted after the first sentence in the first paragraph under the subheading “Flow cytometry” in the Materials and Methods section.

The missing sentence should read: For Glut-1 staining, MDDCs (1 10e5 cells) were incubated with 2.5 µl of Glut1-RBD-GFP construct (Metafora biosystems) for 30 min and fixed with 4% PFA before FACS analysis.

In addition, a reference is omitted from the second sentence of the first paragraph under the subheading “HTLV-1 viral entry is more efficient in DCs that restrict HTLV-1 infection” in the Results section.

The sentence should read: Two proteins, the binding receptor NRP-1 [25] and the fusion receptor Glut-1 [Manel et al., 2003] have been involved in HTLV-1 entry in CD4+ T lymphocytes.

The reference is: Manel N, Kim FJ, Kinet S, Taylor N, Sitbon M, et al. (2003) The ubiquitous glucose transporter GLUT-1 is a receptor for HTLV. Cell 115: 449–459.
