# Supplementary figures and images for: Dendritic cell maturation, but not type I interferon exposure, restricts infection by HTLV-1, and viral transmission to T-cells
Source: PLoS Pathog. 2017 Apr 20;13(4):e1006353. doi: 10.1371/journal.ppat.1006353 (PMC5413061; doi:10.1371/journal.ppat.1006353)

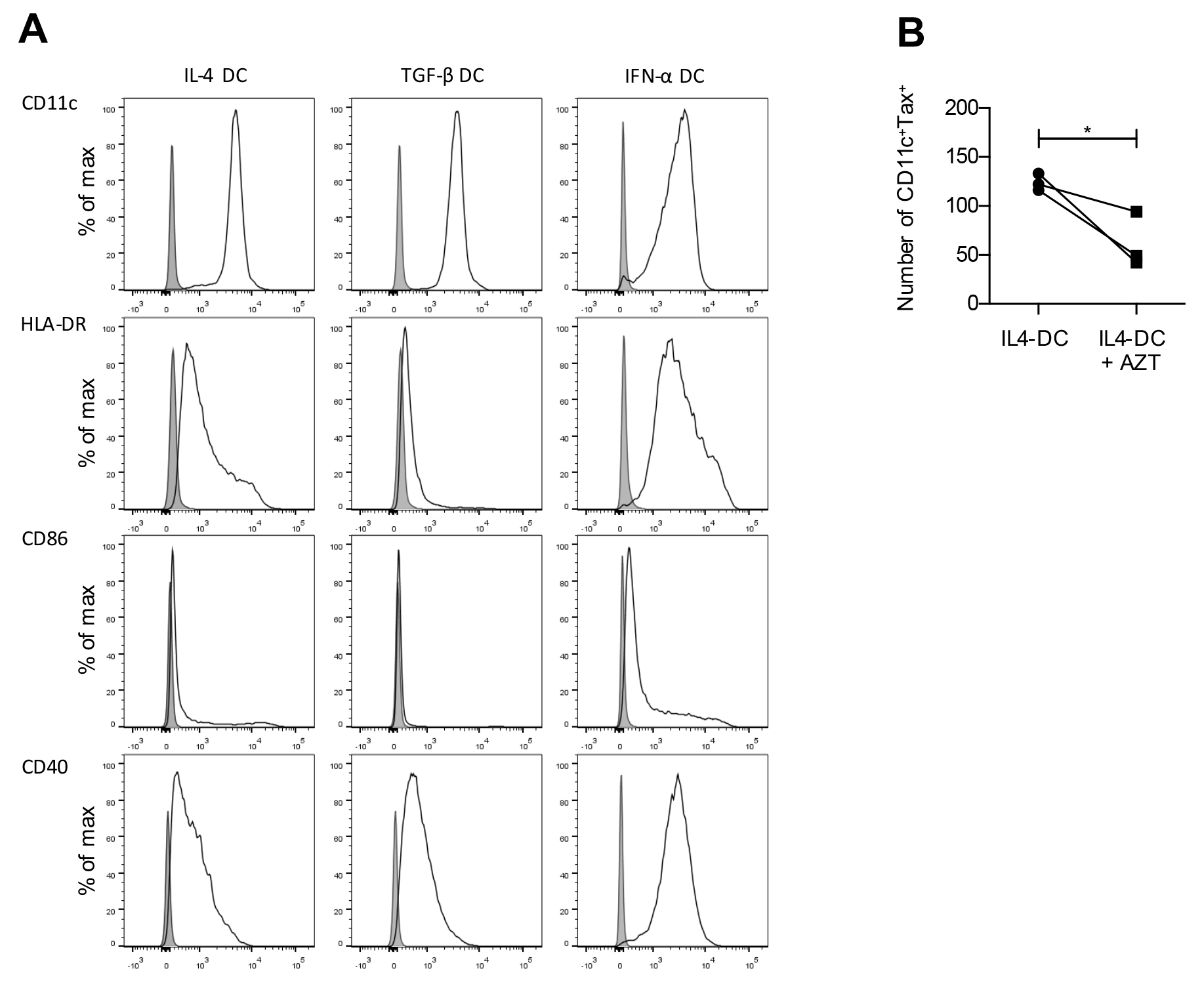

Supplement: S1 Fig — (A) Cells were labeled with fluorochrome-coupled antibodies (black lines) directed against CD11c, HLA-DR, CD83 and CD40. Unstained cells were used as negative controls (black graphs). (B) IL-4 DCs infection was determined by the number of Tax-expressing cells among IL-4 DCs in presence or absence of AZT. Results from 3 independent experiments obtained from different donors are presented. Asterisks indicate statistically significant differences calculated using a paired t-test: *p<0.05. (TIF) [file ppat.1006353.s001.tif]

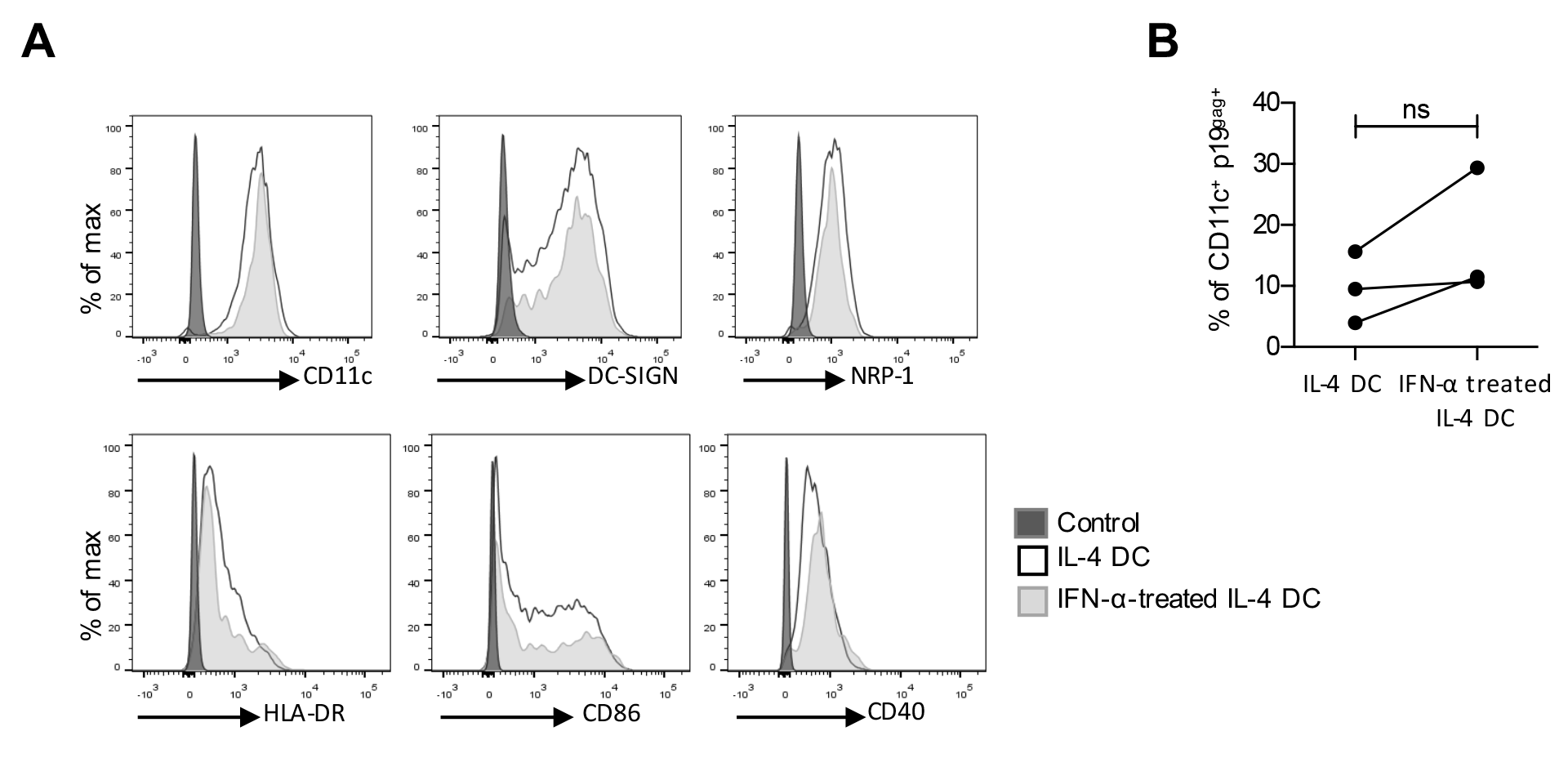

Supplement: S2 Fig — (A) IL-4 DCs were cultured for 18 h in the presence of exogenous IFN-α (2000 IU/ml). Untreated (white histogram) and IFN-α treated IL-4 DCs (light grey histogram) were stained with antibodies directed against CD11c, DC-SIGN, NRP-1, HLA-DR, CD86 and CD40. Unstained cells were used as negative controls (black graphs). (B) Treated and untreated IL-4 DC from the same blood donor were co-cultured with mitomycin-treated C91-PL cells for 3 h and the percentage of p19gag-positive DCs was assessed by flow cytometry. Results are representative of 3 independent experiments performed on different donors. T-test was performed to assess statistically significant differences: ns = non significant. (TIF) [file ppat.1006353.s002.tif]

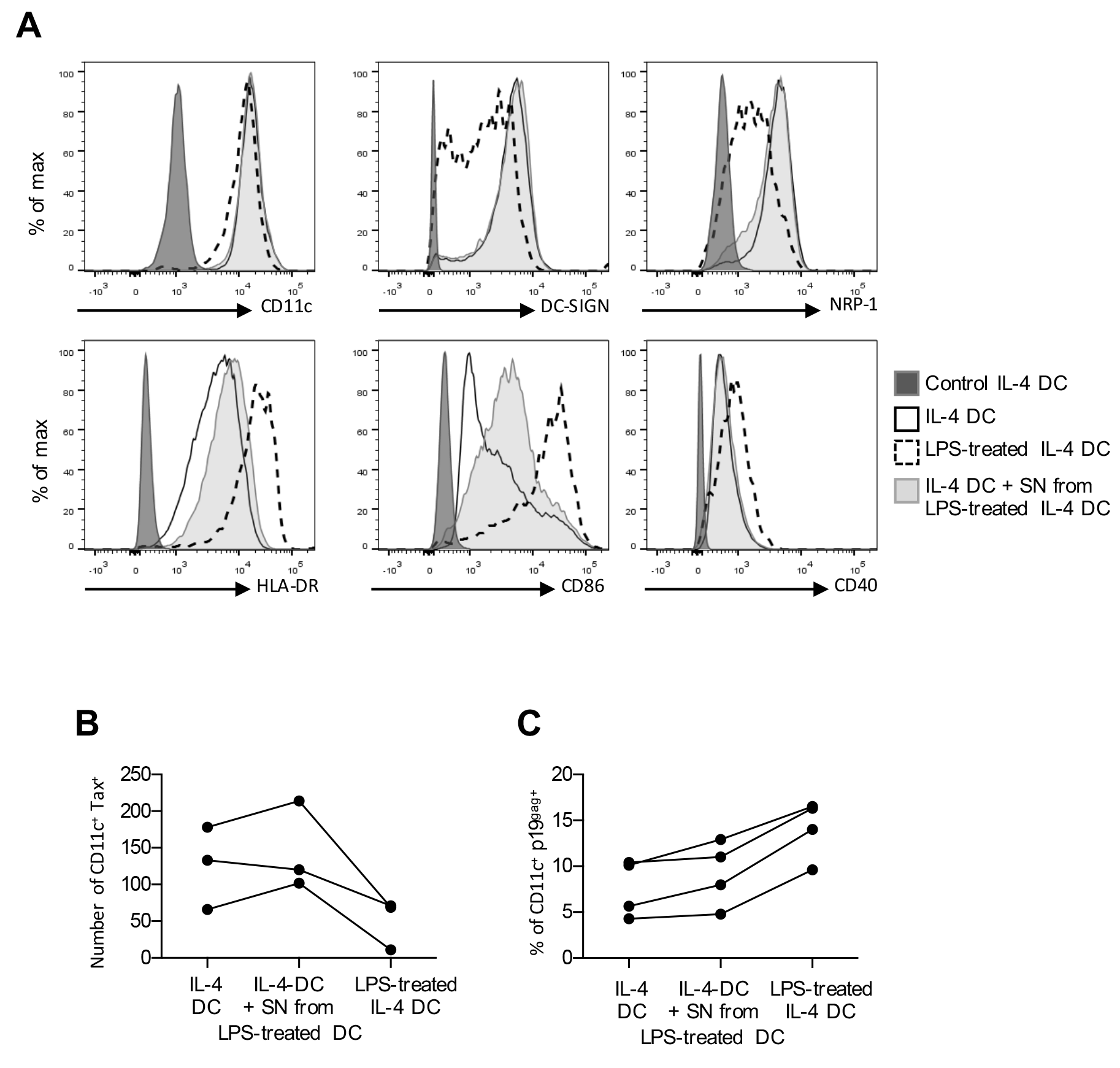

Supplement: S3 Fig — (A) IL-4 DCs from same donor were cultured for 18 h with LPS (black dotted line) or with supernatant from LPS-matured DCs (light grey histogram) or left untreated as a control (black line). Cells were labeled with antibodies against CD11c, DC-SIGN, NRP-1, HLA-DR, CD86 and CD40. Unstained cells were used as negative controls (dark grey histogram). (B and C) IL-4 DCs, IL-4 DCs stimulated with supernatant from LPS-treated IL-4 DCs and LPS-treated IL-4 DCs from the same donor were co-cultured with mitomycin-treated C91-PL cells for 3 h (C) or 3 days (B) and the number of Tax-positive DCs (B) or the percentage of p19gag-positive DCs (C) were assessed by flow cytometry. DCs were discriminated from the C91-PL population by gating on CD11c expression. The results are shown as number of Tax-positive DCs (B) or as percentage of p19gag-positive DCs (C). (TIF) [file ppat.1006353.s003.tif]

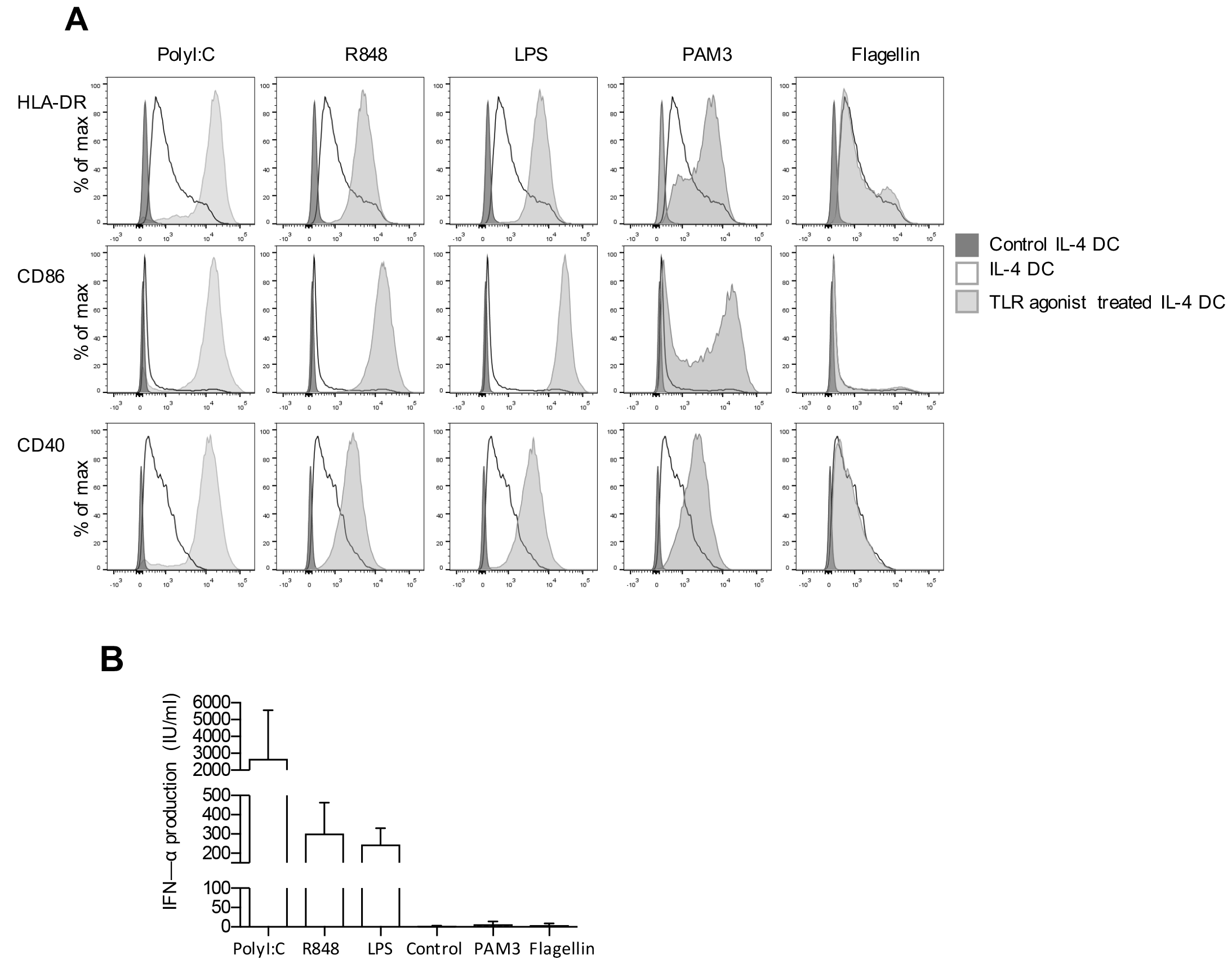

Supplement: S4 Fig — (A) IL-4 DCs were left untreated (white histogram) or were treated for 18 h with the TLR-3 ligand PolyI:C, the TLR-4 ligand LPS, the TLR-7/8 ligand R848, the TLR-2 ligand PAM3CSK4 or the TLR-5 ligand flagellin (light grey histogram). Cells were labeled with antibodies against HLA-DR, CD86 and CD40. Unstained cells were used as negative control (dark grey histogram). (B) Supernatant from untreated IL-4 DC or DC treated with the TLR agonists were collected after 18 h of culture and type I IFN levels measured using HL116 reporter cells expressing ISRE-Luc. (TIF) [file ppat.1006353.s004.tif]

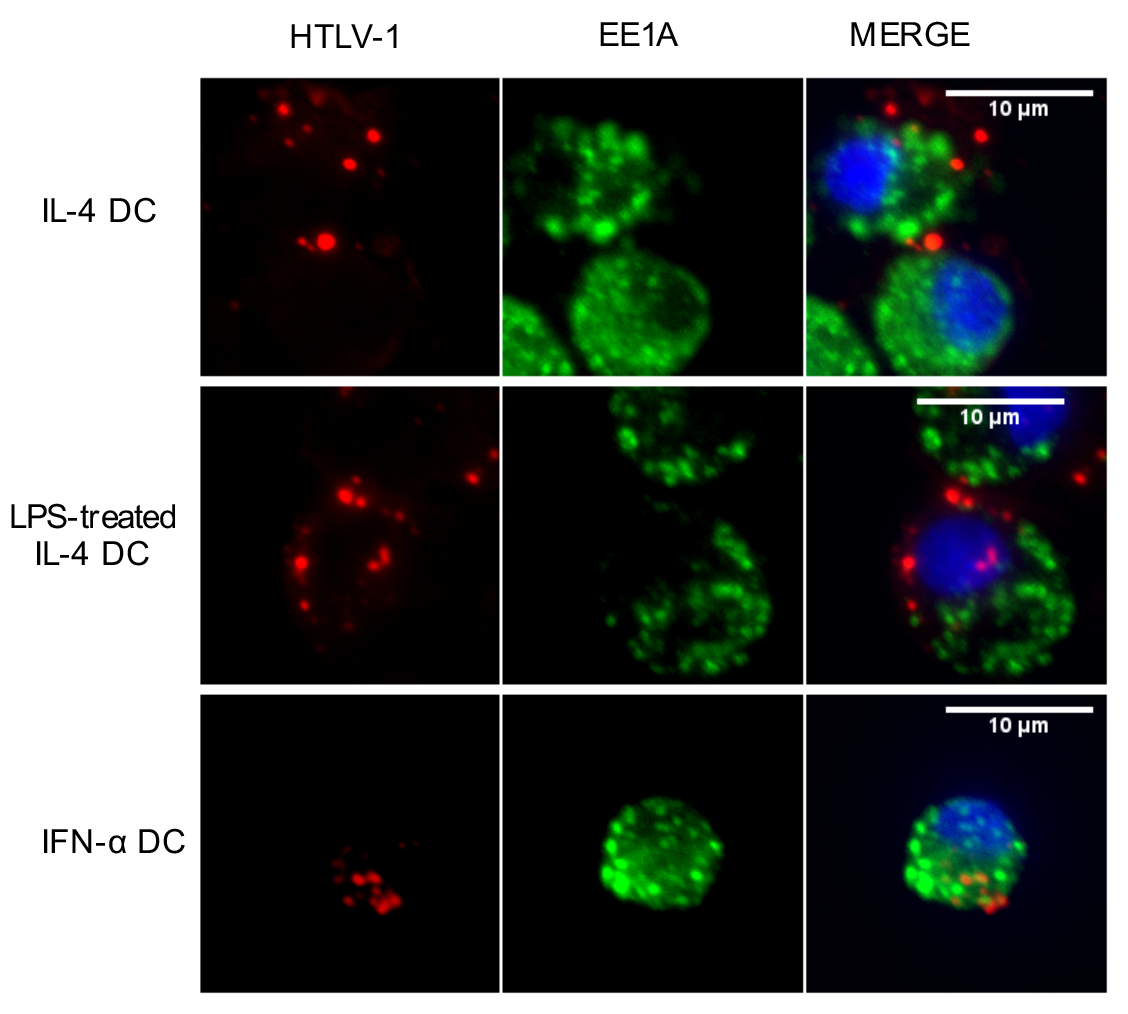

Supplement: S5 Fig — IL-4 DCs, LPS-treated IL-4 DCs and IFN-α DCs were co-cultured with mitomycin-treated C91-PL cells for 3 h, and immunostained for p19Gag (red) and EE1A (green) before analysis by confocal microscopy. Nuclei were counterstained with DAPI (blue). Representative images obtained by confocal microscopy are shown. Scale bars = 10 μm. (TIF) [file ppat.1006353.s005.tif]

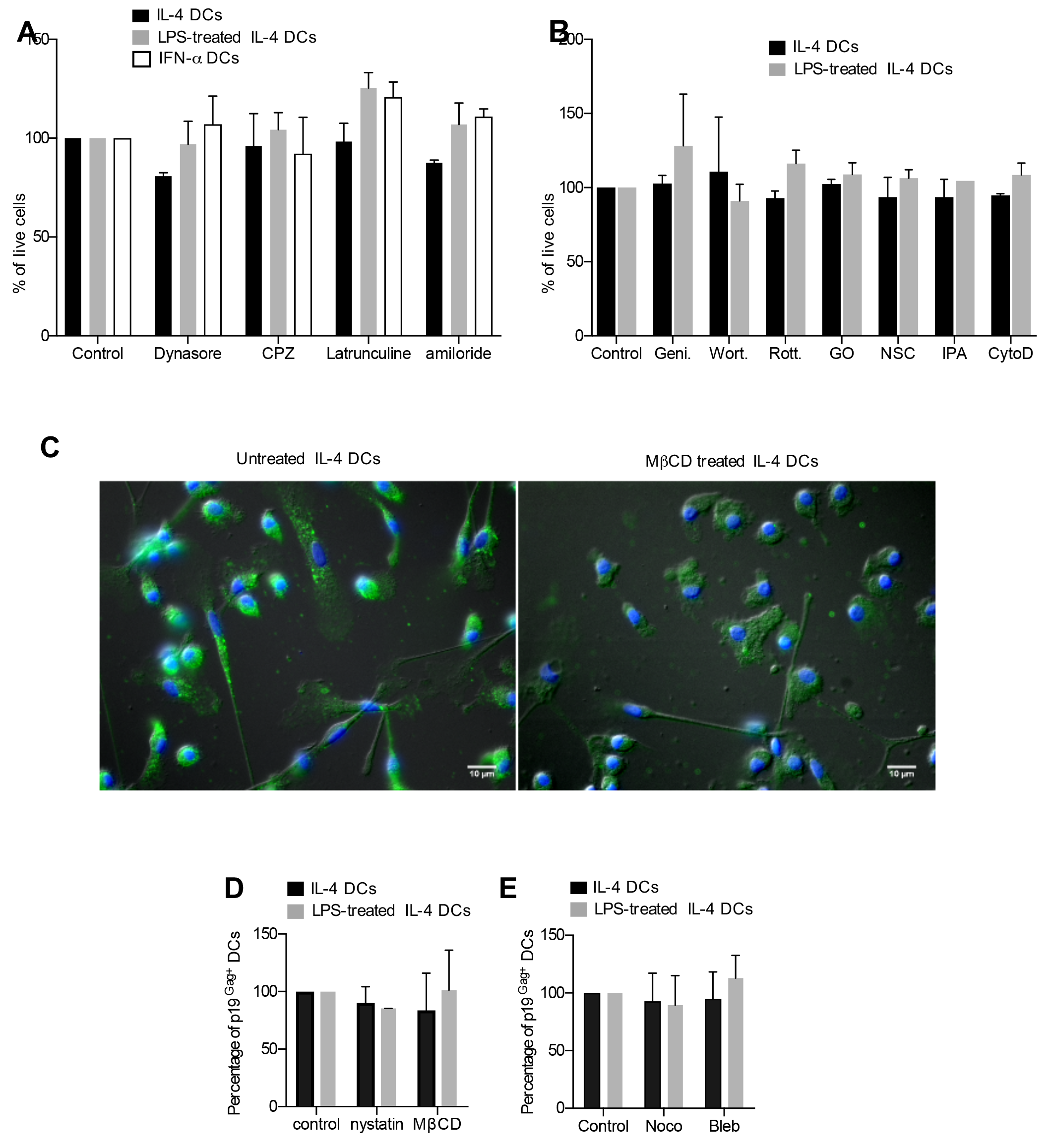

Supplement: S6 Fig — IL-4 DCs (black bars), LPS treated IL-4 DCs (light gray bars) or IFN-α DCs (white bars) were treated for 3 h with (A) dynamin II inhibitors (dynasore, Dyna), clathrin-mediated endocytosis inhibitors (chlorpromazine, CPZ), actin polymerization inhibitors (latrunculin, lat) or macropinocytosis inhibitors (amiloride, ami) or (B) with constitutive macropinocytosis inhibitors (genistein, Gen), PI3K inhibitor (wortmanin, Wort), PKC inhibitors (rottlerin, Rott and Gö6976), Rac-1 inhibitors (NSC23766, NSC), Pak-1 inhibitor (IPA-3, IPA), and actin inhibitor (Cytochalasin D, Cyto D). Cell viability was monitored by FACS using live-dead staining. (C) IL-4 DCs were left untreated (left panel) or treated (right panel) for 3 h with methyl B cyclodextrine (MβCD), fixed and incubated for 15 min with Nile red to stain lipid droplets (green). Nuclei were counterstained with DAPI (blue). Cells were visualized by fluorescence microscopy. (D-E) IL-4 DCs (black bars) or LPS treated IL-4 DCs (light gray bars) were treated for 3 h with nystatin or MβCD to deplete cholesterol (D) or nocodazole (Noco) or blebstatin (bleb) to block microtubule polymerization (E). After treatment, DCs were co-cultured with mitomycin-treated C91-PL cells for 3 h and viral capture determined by flow cytometry using p19gag staining. DCs treated with DMSO were used as controls. The percentage of DCs positive for p19gag was determined for each condition and normalized to that of the untreated control DCs. Results were obtained from 3 independent experiments performed with different blood donors. (TIF) [file ppat.1006353.s006.tif]

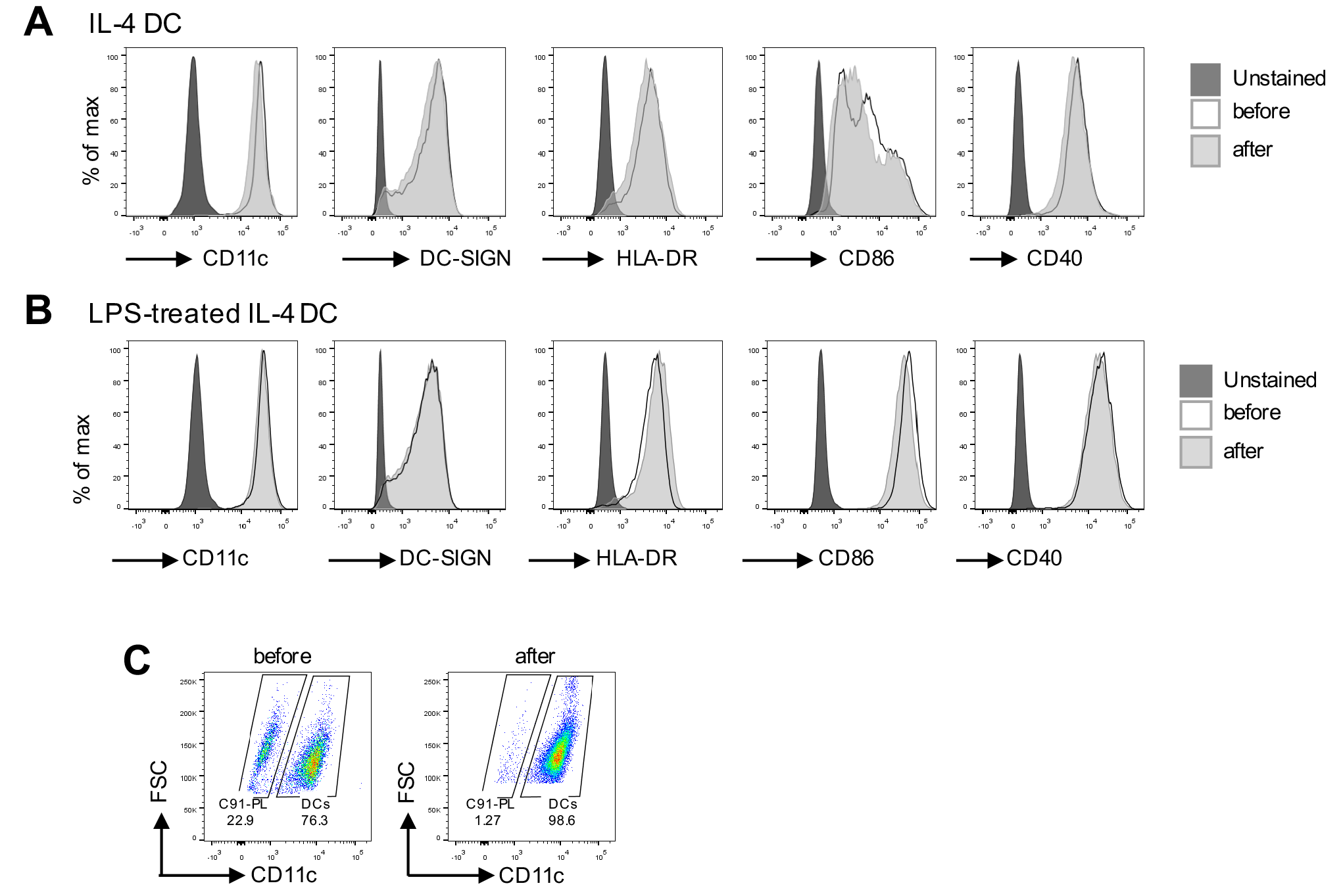

Supplement: S7 Fig — (A) Untreated or (B) LPS-treated IL-4 DCs were exposed to C91-PL for 4 h, left untouched (before, white histograms) or magnetically isolated (after, light grey histograms) and labeled with antibodies against CD11c, DC-SIGN, HLA-DR, CD86 and CD40. Unstained cells were used as negative controls (dark grey histogram). (C) The yield of C91-PL depletion after magnetic isolation of IL-4 DCs was estimated by flow cytometry using anti-human CD11c staining. The percentage of C91-PL cells (CD11c negative population) and the percentage of DCs (CD11c positive population) are indicated on each plot before (left) and after (right) magnetic isolation of DCs. (TIF) [file ppat.1006353.s007.tif]
